# Comparative genomic analysis of azasugar biosynthesis

**DOI:** 10.1186/s13568-021-01279-5

**Published:** 2021-08-23

**Authors:** Hailey E. Beal, Nicole A. Horenstein

**Affiliations:** Department of Chemistry, University of Florida, Gainesville, FL 32611-7200 USA

**Keywords:** 1-Deoxynojirimycin, Azasugars, Biosynthesis, Sequence similarity networks

## Abstract

Azasugars are monosaccharide analogs in which the ring oxygen is replaced with a nitrogen atom. These well-known glycosidase inhibitors are of interest as therapeutics, yet several aspects of azasugars remain unknown including their distribution, structural diversity, and chemical ecology. The hallmark signature of bacterial azasugar biosynthesis is a three gene cluster (3GC) coding for aminotransferase, phosphatase, and dehydrogenase enzymes. Using the bioinformatics platform Enzyme Similarity Tool (EST), we identified hundreds of putative three gene clusters coding for azasugar production in microbial species. In the course of this work, we also report a consensus sequence for the aminotransferase involved in azasugar biosynthesis as being: SGNXFRXXXFPNXXXXXXXLXVPXPYCXRC. Most clusters are found in *Bacillus* and *Streptomyces* species which typically inhabit soil and the rhizosphere, but some clusters are found with diverse species representation such as *Photorhabdus* and *Xenorhabdus* which are symbiotic with entomopathogenic nematodes; the human skin commensal *Cutibacterium acnes,* and the marine *Bacillus rugosus* SPB7, a symbiont to the sea sponge *Spongia officinalis*. This pan-taxonomic survey of the azasugar 3GC signature may lead to the identification of new azasugar producers, facilitate studies of their natural functions, and lead to new potential therapeutics.

**Supplementary Information:**

The online version contains supplementary material available at 10.1186/s13568-021-01279-5.

## Key points

The enzyme similarity tool suite has been used to analyze azasugar biosynthesis.

A wide range of genera are identified that contain new potential azasugar producers.

A consensus sequence for aminotransferases involved in azasugar biosynthesis is reported.

## Introduction

**Fig. 1:**
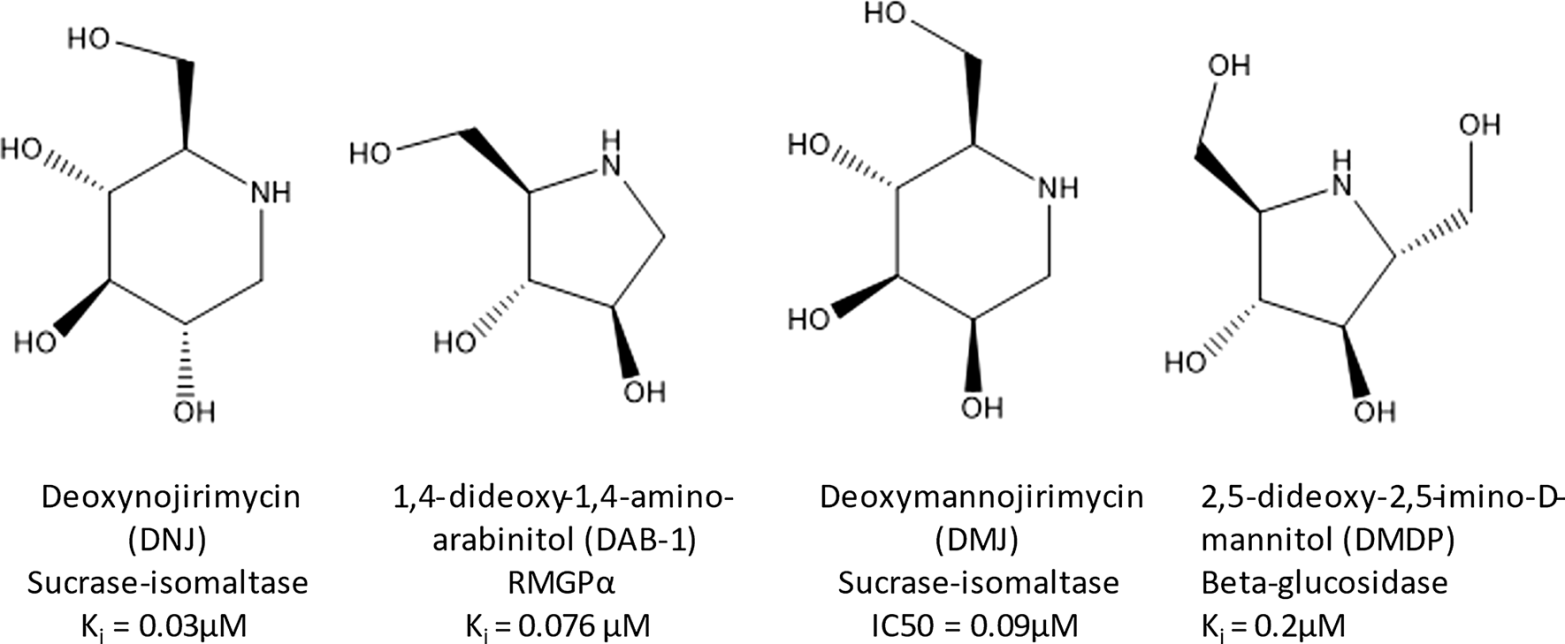
Microbial azasugars and examples of glycosidases they inhibit

Azasugars are analogs of monosaccharides with nitrogen replacing the ring oxygen found in a conventional sugar and are produced by bacterial and plant species(Asano 2003; Shibano et al. 2004; Konno et al. 2006; Song et al. 2009; Zhang et al. 2019; Gao et al. 2016). They (Fig. 1) are well known as glycosidase inhibitors and have served as a platform for the development of drugs used for diabetes and lysosomal storage diseases (Asano 2003). Their ability to target glycosidases is presumably linked to their natural function(s), yet to be established. Deoxynojirimycin (DNJ) has been shown to be toxic towards caterpillars (Konno et al. 2006) and reduce biofilm of *Streptococcus mutans* (Islam et al. 2008) hinting at a few possible natural roles DNJ and other azasugars might play. The most common plant sources are the mulberry bush (*M. alba*) and the dayflower (*C. communis*), whose leaves contain DNJ, galactonorjirimycin (Gal-DNJ), 1,4-dideoxyaminoarabinitol (DAB-1) and 2,5-dideoxy-2,5-imino-d-mannitol (DMDP) (Shibano et al. 2004; Konno et al. 2006; Song et al. 2009; Kim et al. 1999; Nakagawa et al. 2010). The limited biosynthetic information available for plants suggests that plants and microbes produce these azasugars through different routes. While both use glucose as a precursor, *C. communis* aminates the C1 position and oxidizes at the C5 position allowing for a C1/C5 cyclization (Shibano et al. 2004). Microbial biosynthesis, however, is a head-to-tail cyclization where the C2 keto group of fructose-6-phosphate is aminated and the C6 hydroxyl is oxidized, leading to a C2/C6 cyclization (Hardick 1992; Clark et al. 2011). Because of the much greater number of complete microbial genomes relative to plants, and the ease of genetic manipulation, we focus on microbial azasugar production in this report.

**Fig. 2:**
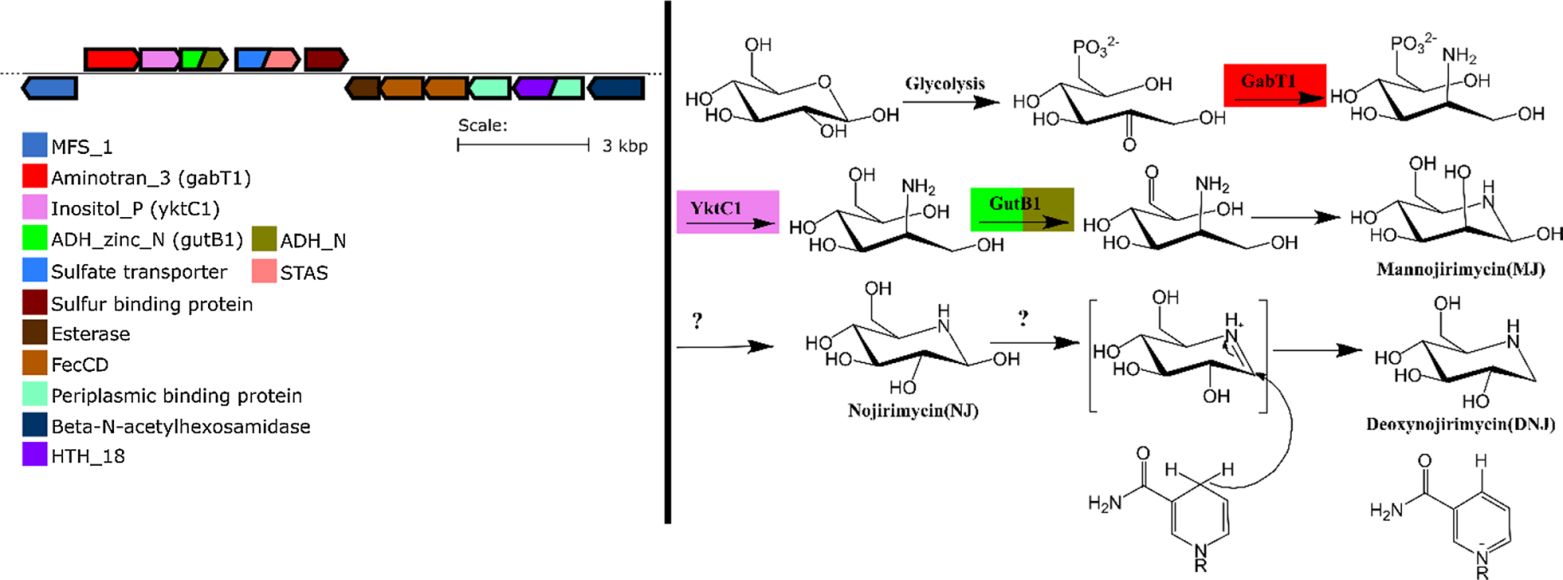
DNJ biosynthesis in *B. velezensis* (formerly *B. amyloliquefaciens* FZB42). Genome Neighborhood Diagram from *B. amyloliquefaciens* FZB42 adapted from EST-EFI tool (https://efi.igb.illinois.edu/efi-gnt/) not pictured upstream of the MFS transporter are tRNA ORFs

Foundational work concerning the biosynthesis of azasugars in bacteria included feeding experiments using site specific isotope labeling in precursor sugars for *Bacilli* (Hardick et al. 1992) and identification of genes coding for DNJ biosynthetic enzymes in *Bacillus amyloliquefaciens* FZB42 (Clark et al. 2011), reclassified as *Bacillus velezensis* (Fan et al. 2017), *B. subtilis* MORI 3K-85 (Kang et al. 2011), and biosynthetic genes involved in production of 1,4-dideoxy-1,4-aminoarabinitol (DAB-1) in *Chitinophaga pinensis* (Nuñez and Horenstein 2019). The key biosynthetic machinery (Fig. 2) starts with an aminotransferase responsible for transferring an amino group to a phospho-ketose. Removal of the phosphate group via a phosphatase, followed by oxidation at the newly unmasked hydroxyl group by an aminopolyol dehydrogenase yields an intermediate oxo-aminopolyol (Wu and Horenstein 2013). Spontaneous cyclization with the amino group attacking the newly formed carbonyl at O6, produces the azasugar. The three genes for the aminotransferase, phosphatase and dehydrogenase enzymes used in the biosynthesis are found clustered together in all known azasugar producers examined thus far (Clark et al. 2011; Nuñez and Horenstein 2019) and we refer to them as the 3GC. In the case of DNJ biosynthesis, two more steps are needed to complete the work of the 3GC, namely the *manno*-configuration needs to be epimerized to the *gluco*-configuration at C2, and then the 1-OH group is reduced to the 1-deoxy form. Interestingly, these enzymes do not appear to be clustered with the 3GC and have not yet been identified. For example, the two genes in Fig. 2 which appear directly after the 3GC in *Bacillus velenzensis* FZB42 (RBAM_002070 and RBAM_002080) code for putative anion/sulfate transporter and a sodium bile acid symporter proteins respectively. These have been BLASTed against the PDB database and confirmed as similar to respective transporters and therefore are not likely to be part of the azasugar synthetic machinery. However, we hypothesize that the MFS transporter (RBAM_002060) may be involved in azasugar transport, as it is commonly found associated with 3GCs. Given that known azasugar producers feature the aforementioned three gene cluster (3GC), we suggest that this signature may be useful for identification of new bacterial producers, new azasugars, and possibly provide new sources of carbohydrate active enzymes that are able to perform stereoselective chemical transformations such as amination and regioselective redox chemistry.

We originally identified 3GCs by using BLAST to search for aminotransferases and manually searching through their immediate genomic neighborhoods for phosphatase and dehydrogenase coding ORFs, a strategy used to successfully identify the 3GC in *B. amyloliquefaciens* FZB42 as well as the DAB-1 3GC in *C. pinensis* DSM 2588 (Clark et al. 2011; Nuñez and Horenstein 2019). This method was also recently used to identify and compare potential DNJ producers in the *Bacilli* genus (Lee et al. 2021). Another method that has been used to find a 3GC is through PCR screening using degenerate primers, which was employed to identify the 3GC in *Bacillus amyloliquefaciens* 140N, a species isolated from fermented soybean (Seo et al. 2013). Although these methods work, the two limitations are that the BLAST approach is tedious, and the PCR approach will work best for closely related species. We therefore sought an approach that might offer a more global perspective and provide a means of identifying interesting enzyme activities within putative azasugar operons broadly distributed across microbial species. Bioinformatic suites such as the Enzyme Function Initiative (EFI) tools (Gerlt et al. 2015; Zallot et al. 2018) offer a comparative approach to identification and analysis of 3GCs within different organisms. The EFI tools enable *en masse* comparative analyses of genomic sequence data. The Enzyme Similarity Tool (EST) creates sequence similarity networks (SSN), in which enzymes from different organisms are clustered based on their degree of sequence identity. SSNs are a powerful tool to identify new groups of enzymes within a family and have been used for family-wide activity profiling of halogenases (Fisher et al. 2019) and *Streptomyces* cytochrome p450s, (Rudolf et al. 2017) to name a few examples. These SSNs can then be further processed through the Genome Neighborhood Tool (GNT) to produce genome neighborhood networks (GNN). A GNN identifies open reading frames proximate to a query node within a SSN to provide a graphical representation of the enzymes associated with the query; thus, aiding in identification of biosynthetic operons. This technique has been used in broad, large scale studies of entire families of proteins such as proline racemases (Zhao et al. 2014) as well as for the exploration of bacterial genomes for the discovery of new natural products such as enediynes (Rudolf et al. 2016). In the study we report here, we utilized the EST and GNT to analyze microbial sequence data for the incidence of the azasugar 3GC signature. The data generated from these analyses has helped us identify potential azasugar producers and may facilitate understanding of azasugars in the context of evolution, molecular diversity, function, and chemical ecology.

## Methods

### Computational/database tools

Data from reported bacterial protein sequences from Uniprot were analyzed with the EFI tools for creation of SSNs and GNNs (https://efi.igb.illinois.edu/). Data were curated and visualized locally using Cytoscape (Shannon et al. 2003; Cline et al. 2007).

### SSN generation

The aminotransferase, RBAM_RS01020(GabT1) and the aminopolyol dehydrogenase (APD), RBAM_RS01030(GutB1) from the genomic sequence NC_009725.2 obtained for the azasugar producer *B. velezensis* FZB42, were used to construct two SSNs. These were submitted through the single sequence input option with an expect score of 10^–5^ and a maximum of 10,000 hits generated to allow for all similar sequences to be analyzed. From there, alignment score thresholds of 110 and 80 were chosen which correlates to approximately 40% identity to the aminotransferase, GabT1, and dehydrogenase, GutB1, respectively (Additional file 1: Figure S3). 40% identity was chosen to create a distinguishable cluster pattern of similar protein sequences and to avoid the “twilight zone”, (Rost 1999) where non-similar proteins tend to have 15–30% identity amongst themselves (Joshi and Xu 2007). To create a more stringent network, the alignment scores of 140 and 95 were chosen which correlates to approximately 50% identity (Additional file 1: Figure S3). Lastly, alignment scores (GabT1, GutB1) correlating to 55% (145,110), 60% (150,125) and 70% (180,145) identity were generated to have a broader understanding of the clustering of enzymes with the increase of stringency of alignment scores (Additional file 1: Figure S4 and S5).

### Using SSNs to identify 3GC containing genomes

**Fig. 3:**
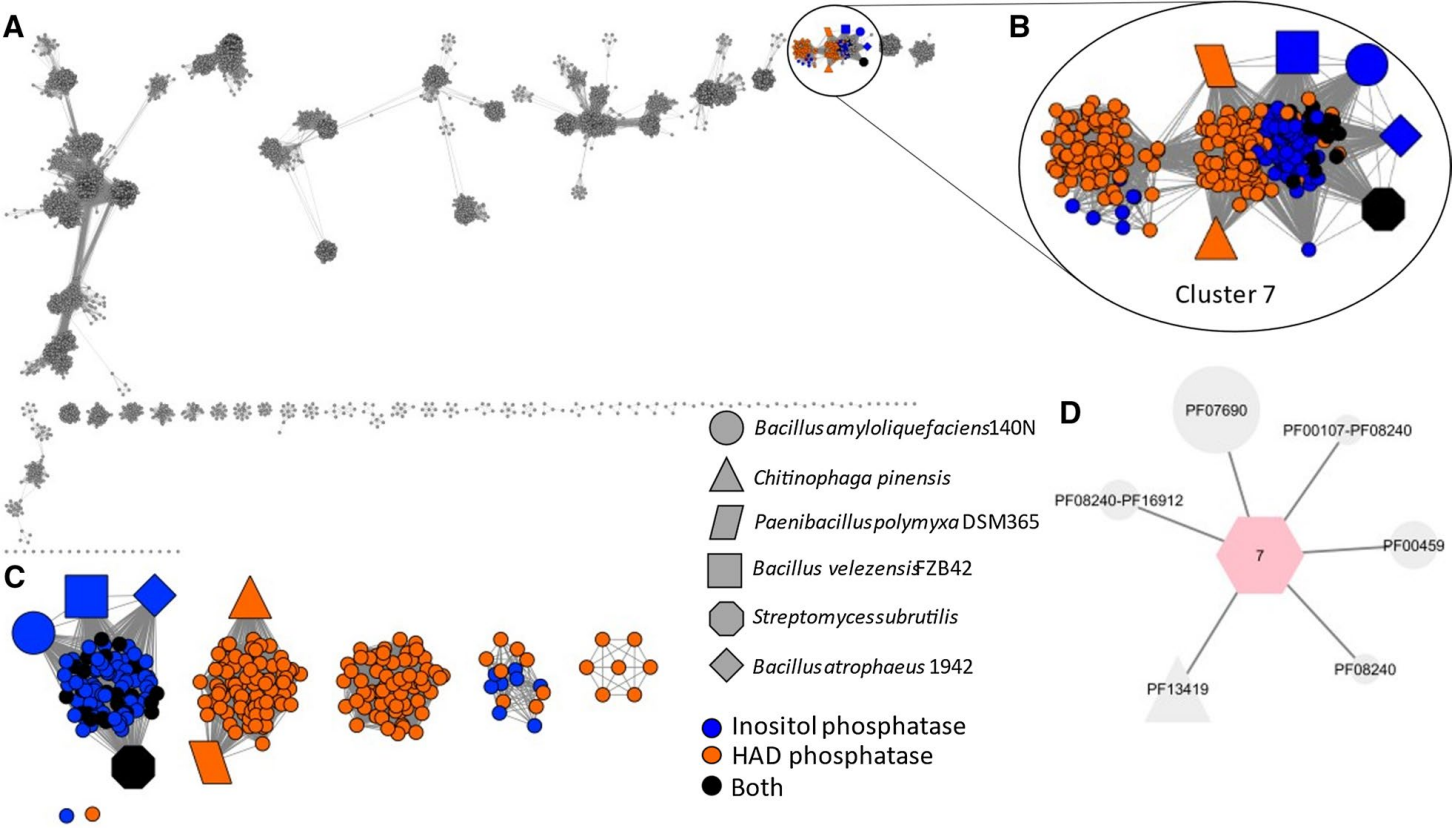
**A** Aminotransferase SSN with an alignment score threshold of 40%. The nodes with neighboring dehydrogenase and phosphatase genes are colored based on the PFAM of phosphatase. **B** SSN containing only the 3GC nodes. **C** Aminotransferase SSN with an alignment score threshold of 50% containing only the 3GC nodes. **D** GNN for cluster 7; Known azasugar producers are assigned the following corresponding shapes: Parallelogram: *P. polmyxa* DSM365, Triangle: *C. pinensis* DSM2588, square: *B. velezensis* FZB42, diamond: *B. atrophaeus* 1942, and large circle: *B. amyloliquefaciens* 140N, Octagon: *S. subrutilis*

**Fig. 4:**
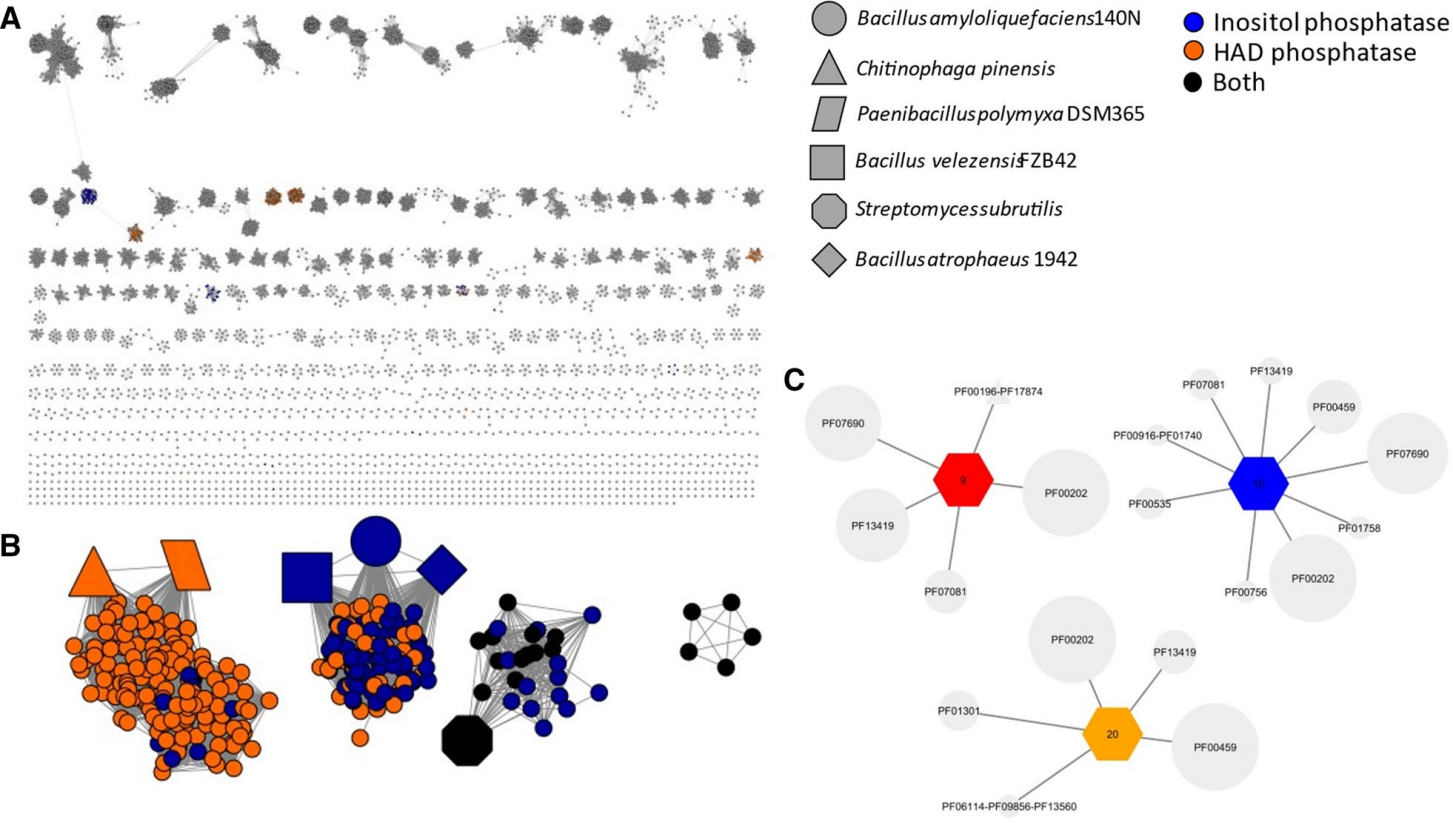
**A** Dehydrogenase SSN with alignment score thresholds corresponding to 40% identity. **B** Dehydrogenase SSN with only the 3GC nodes. **C** GNN for clusters with known azasugar representation: 9,10 and 20. Polygons identify species as defined for Fig. 3

The SSNs generated above were processed through the Genome Neighborhood tool (GNT) to create both Colored SSNs and Genome Neighborhood Networks(GNNs). These were generated with a co-occurrence of 20% and a neighborhood size of ± 3 ORFs. All networks were formatted using the Prefuse Force directed OpenCL layout (Shannon et al. 2003) (Figs. 3 and 4). For comparative purposes, the nodes representing either aminotransferase or dehydrogenase enzymes within a known azasugar producer were highlighted. The following symbol code was used: triangle, *C. pinensis* DSM7; parallelogram, *P. polymyxa* DSM365; square, *B. velezensis* FZB42; circle, *B. amyloliquefaciens* 140N; diamond, *B. atrophaeus* 1942; octagon, *S. subrutilis* ATCC 27467 (Schmidt et al. 1979; Hardick et al. 1992; Clark et al. 2011; Gibbons et al. 2011; Seo et al. 2013; Nuñez and Horenstein 2019).

The SSN for the aminotransferase at 40% identity gave us a cluster, cluster 7, in which known azasugar producers group. The protein families within cluster 7 of the GNN corresponding to the dehydrogenase and phosphatase activities were identified as: PF00107 and PF08240, PF16912 for the dehydrogenase; and PF13419 and PF00459, for the phosphatase. The SSN for the dehydrogenase at 40% identity contained three clusters which contained known azasugar producers, clusters 9, 10 and 20. The GNN for these clusters gave us the protein families: PF00202 for the aminotransferase and PF13419 and PF00459 for the phosphatase. However, the phosphatase for *C. pinensis* was a member of the PFAM PF12710, which was also included in our subsequent inquiry.

Using the protein families collected from the GNNs, a query on the colored SSNs was done to find all nodes which contained at least one of each PFAM for each of the required enzyme activities required for the pathway. These were highlighted and colored coded based on which PFAM of the phosphatase they had, either HAD hydrolase or inositol phosphatase. For visualization purposes, the subset of nodes with a 3GC were created (Figs. 3C, 4C).

The two sets of 3GC containing SSNs (Figs. 3C and 4C) were tabulated in Additional file 1: Table S1, including organism name, source, gene names for aminotransferase and dehydrogenase activities, and SSN cluster ID.

## Results

### Aminotransferase and dehydrogenase SSNs

Figure 3A presents the aminotransferase SSN using a minimum 40% amino acid identity threshold. We found that known azasugar producing organisms with genomic information available group into a single cluster, cluster 7, for the aminotransferase SSN (Fig. 3B). Cluster 7 had a total of 315 nodes, 251 of them containing 3GCs. All 3GC-containing nodes on this SSN grouped into cluster 7. When the amino acid identity threshold was increased to 50% (Fig. 3C) we observed the 3GC-containing nodes separate into five distinct clusters and two singletons (single-membered clusters). Two of the five clusters harbored known azasugar producing species. More stringent SSNs with alignment scores correlating to 55, 60, and 70% identity were produced (Additional file 1: Figure S4, S5) and show the aminotransferases belonging to *P. polymyxa* and *C. pinensis* separated into different clusters between 60 and 70% identity. *S. subrutilis’* aminotransferase deviated from the *Bacilli* azasugar producer’s aminotransferases at 70%.

**Fig. 5:**
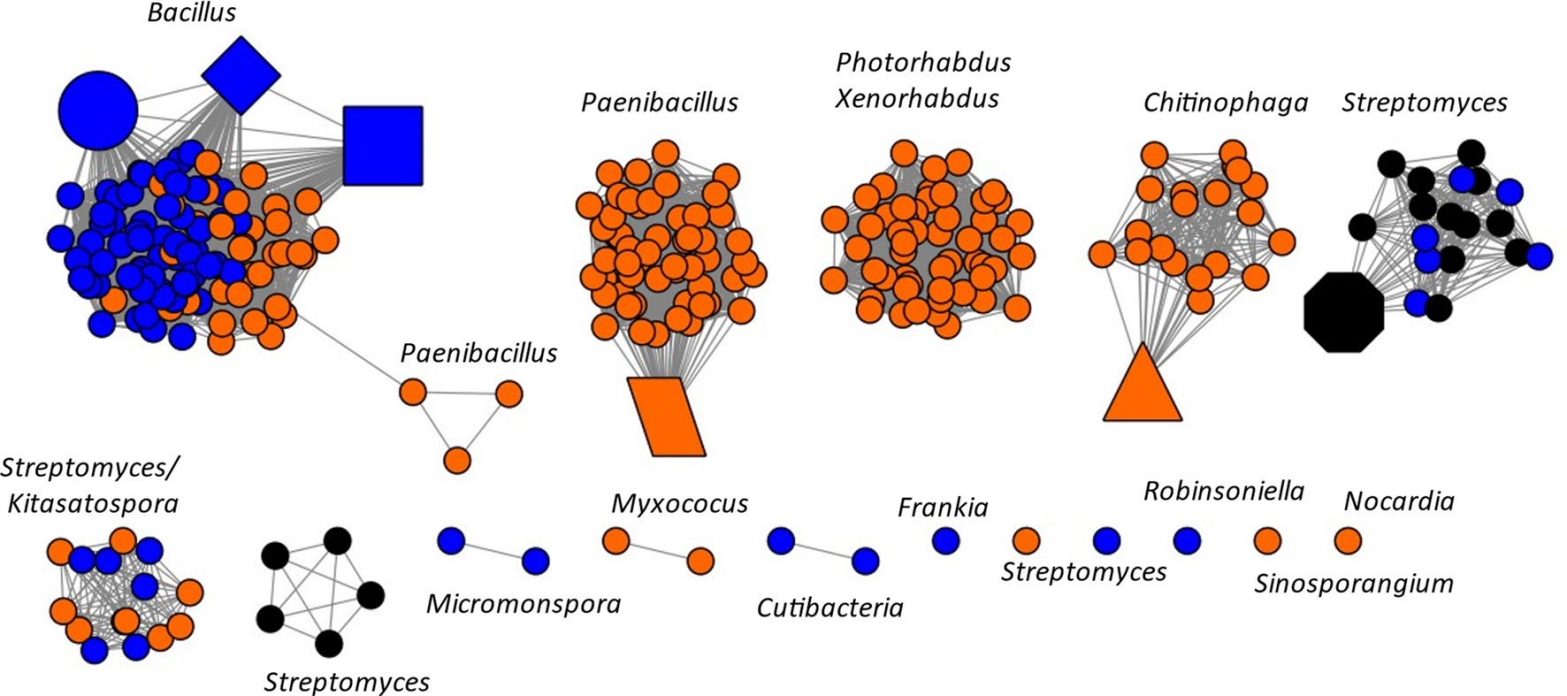
**A** Aminopolyol dehydrogenase SSN with an alignment score threshold of 50% with only 3GC containing nodes. Color coding based on type of phosphatase found within 3GC. Clusters are labeled based on what genera is 75% or more of cluster, if this condition was not met, the top two genera are listed. Polygons identify species as defined for Fig. 3

We observed more clusters for the dehydrogenase SSN at 40%, compared to the aminotransferase, indicating there is a higher level of variability amongst putative azasugar dehydrogenases than for aminotransferases. The dehydrogenase SSN at 40% has four clusters, three with known azasugar representation. As the alignment score threshold increases to 50%, the SSN expanded to ten clusters and seven singletons (Fig. 5). If a cluster contained at least 75% of the same genera, it was labeled with the genera name; if this condition was not met then the two most populous genera were listed (Fig. 5). At 50% sequence identity for the dehydrogenase, we see the known azasugar producers separate based on genera: the *Bacilli* cluster, while *Streptomyces subrutilis, Paenibacillus polymyxa*, and *Chitinophaga pinensis* deviate into separate clusters.

There were 251 putative producers found on the aminotransferase SSN and 268 putative producers found on the dehydrogenase SSN which had significant overlap, creating a total of 296 unique hits including six known producers. (Additional file 1: Table S1) The genera of the putative producers were tabulated and listed in Table 2 if five or more nodes of a specific genera were identified in the SSNs.

To augment the results of our SSNs, we used pBLAST for each enzyme of the 3GC for *B. velezensis* with the corresponding enzymes in each of the other five known azasugar producers (Table 1). As expected, the aminotransferases are more similar than dehydrogenases. From the table, phosphatases are observed to be variable in sequence identity; the Bacilli producers have similar phosphatases, but *Streptomyces*, *Paenibacillus* and *Chitinophaga* are different. Though the phosphatase BLAST score for *P. paenibacillus* suggests a higher similarity, this is not the case because the query coverage is only 20%. Further, the phosphatase enzymes of the 3GCs are represented by multiple and diverse PFAMs. (Additional file 1: Figures S6 and S7) The *Bacillus* and *Streptomyces* strains’ phosphatases are annotated as inositol-phosphate phosphatases (PFAM: PF00459), while the *Chitinophaga* and *Paenibacillus* strains’ phosphatases are annotated as haloacid dehalogenase (HAD)-like hydrolases (PFAM: PF12710, PF07081, and PF13419). Because we wanted to explore how the sequences of producers related to each other, the phosphatase sequences were not practical because they were too dissimilar for all to be included in one SSN.

**Table 1:** pBLAST scores of the five known azasugar producers against *Bacillus velezensis* FZB42 with correlating E-values

Strain	Aminotransferase % ID	Query cover	E-value	Dehydrogenase % ID	Query cover	E-value	Phosphatase % ID	Query cover	E-value
*Bacillus amyloliquefaciens* 140 N	99.29	100%	0.0	98.56	100%	0.0	97.15	100%	0.0
*Bacillus atrophaeus* 1942	92.89	100%	0.0	86.21	100%	0.0	88.61	100%	0.0
*Streptomyces subrutilis* ATCC 27,467	60.77	98%	0.0	32.94	98%	5E−57	N/A	N/A	N/A
*Paenibacillus polymyxa* DSM 365	48.11	96%	9e−137	32.22	92%	2e−59	66.67	20%	0.017
*Chitinophaga pinensis* DSM 2588	46.57	97%	2e−134	32.18	97%	3e−59	N/A	N/A	N/A

### Identification of an aminotransferase consensus sequence

A multiple sequence alignment for the six known azasugar producer’s aminotransferases was generated through EMBL-EBI Clustal (Additional file 1: Figure S9). A consensus sequence was created through EMBOSS Con. This consensus sequence was then analyzed using InterPro which led to placement of the sequence in PIRSF000521 (Additional file 1: Figure S10). The domain architecture was PFAM PF00202, aminotran_3, classified as a 4-aminobutyrate/lysine/ornithine transaminase. From this analysis we identified a 19 amino acid span between positions 145–164 that was not conserved in PF00202, having the sequence AFRREPFPpqIxSfgLQVPD. We then asked if this broadly unconserved sequence might be diagnostic for aminotransferases involved in azasugar production.

**Fig. 6:**
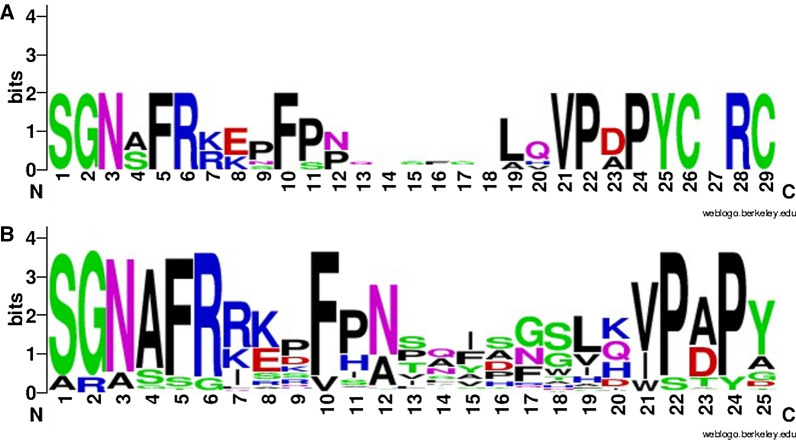
**A** WebLogo sequence of all known azasugar producers (corresponding to amino acids 145–170 in *B. velezensis* FZB42). **B** WebLogo sequence of all known and putative azasugar producers (corresponding to amino acids 145–170 in *B. velezensis* FZB42)

Starting with this 19 amino acid span as the focus, we performed a multiple sequence alignment of the aminotransferases for all known and putative azasugar producers and visualized this region using WebLogo (weblogo.berkley.edu) (Fig. 6). The WebLogo generated for all known 3GC aminotransferases was simplified into the consensus sequence SGNXFRXXXFPNXXXXXXXLXVPXPYCXRC. When this consensus sequence was analyzed using the NCBI conserved domain search to check for any recognition of sequence, no conserved domain was recognized. When we used this sequence as a pBLAST query against the entire NR database, we identified 214 aminotransferase hits at or below a threshold E-value of 0.11 that were part of a 3GC based on comparison to the SSN results, and manual inspection. A small number of the blast hits (9, 4% of total hits) were not part of a 3GC.

## Discussion

Despite the fact that microbial production of azasugars been known since the 1960s, relatively few species have been identified as producers of these compounds via traditional techniques of fermentation and compound isolation. While this wet chemistry is the gold standard for confirmation of natural product production, this could be the last step in a workflow that starts with a more efficient screening approach. Starting from the observation that thus far, known azasugar production minimally involves three key enzyme activities (aminotransferase, dehydrogenase, phosphatase) we hypothesized that this is a general feature of the biosynthetic pathway in other organisms, and that we can find them based on sequence information from known azasugar producers. In the work we report here, we applied sequence similarity networks (SSNs) as a primary tool to identify organisms that had 3GCs. Coincidence of the genes coding for these activities is probative for the pathway, so we utilized genome neighborhood networks to color code the SSNs to facilitate identification of 3GCs.

### Sequence similarity networks

A sequence similarity network allows one to identify specific enzymes that are related by their level of sequence similarity. Unlike BLAST that compares one sequence to others in a database, the SSN approach starts with a single sequence, but involves comparison between all members of the set. Similar to BLAST, this method is still lacking genomic context, meaning that one would have to manually inspect the sequence data to identify flanking genes and evaluate if they were the required activity for the pathway of interest. This can be avoided by using a colored SSN, a function of the GNT. A colored SSN can now add genomic context to each node within an SSN, and this can be queried to identify nodes with neighboring genes of interest.

The aminotransferase SSN (Fig. 3) appears to be more conserved than the dehydrogenase at the same stringency of sequence identity. The putative azasugar producers with the 3GC characteristic are found exclusively in cluster 7 of the aminotransferase SSN at 40% sequence identity. (Fig. 3) The dehydrogenase SSN had four clusters with known and putative azasugar producers; three of the clusters had at least one known azasugar producer within. One reason that clusters lacking known azasugar producers are of interest is because their sequences are somewhat diverse compared to the known producers. The greater similarity of aminotransferases suggested that they may be the best gene within a 3GC for development of a consensus sequence. Based on the dehydrogenase SSNs we observed greater diversity in these enzymes which one could consider as a predictive tool for the specificity of dehydrogenases, and the identity of the azasugar being produced. Yet enzymes in different clusters can produce the same azasugar (Figs. 3C and 4B). Because the database of known producers is extremely small, we cannot yet use the SSN to predict the azasugar produced. *C. pinensis* is the only known azasugar producer on our list which produces DAB-1 instead of DNJ. Yet it does cluster with some known DNJ producers under the parameters of our analyses. In our analyses we found considerable variability in the sequences and PFAMs found for phosphatases that were part of 3GCs, resulting in the requirement to create multiple SSNs to include all known azasugar producers. (Additional file 1: Figure S6 and S7). Therefore, we did not consider the phosphatase as a useful signature for azasugar production.

We found that within individual 3GCs gene order varied in two ways. The *Bacilli* DNJ producers (Fig. 2) have the gene order aminotransferase, phosphatase, and dehydrogenase, whereas the gene order of *C. pinensis’* and putative producers in *Chitinophaga* and *Paenibacillus* genera was aminotransferase, dehydrogenase and phosphatase. In parallel, these two groups also had different phosphatase families. The *Bacilli* phosphatase was annotated as being in the inositol phosphatase family while the *Chitinophaga/Paenibacillus* group featured phosphatases from the HAD hydrolase family.

Some of the nodes within the SSNs were not associated with 3GCs, but might be of interest for work on related classes of compounds. One example is the aminotransferase ValM from *Streptomyces hygroscopicus subsp. Jinggangensis* 5008, which was identified in cluster 7 of the 40% aminotransferase SSN. *S. hygroscopicus* is a known producer of the aminocyclitol validamycin, a potent inhibitor of trehalase (Bai et al. 2006; Fan et al. 2013; Tang et al. 2017). ValM is part of the biosynthetic pathway of validamycin and is 50% similar to GabT1 in *B. velezensis* by pBLAST. As a sign of the difference between the aminocyclitol pathway vs. the azasugar pathway, the neighboring dehydrogenase ValN is only 23% similar to GutB1 in *B. velezensis*, was not on the GutB1 SSNs, and lacked a phosphatase.

### A consensus sequence identifies three gene clusters

We identified the consensus sequence SGNXFRXXXFPNXXXXXXXLXVPXPYCXRC for aminotransferases involved in azasugar production. This allowed us to identify 214 BLAST hits that were part of a 3GC, 128 of which were not previously identified in an SSN. (Additional file 1: Table S1) The realization of additional 3GCs provides a clear demonstration of the utility of using a BLAST of the aminotransferase consensus sequence to augment the results derived from the SSN study. It is also important to note that the consensus BLAST results missed some aminotransferases that did appear in the SSN that were also associated with 3GCs, so both approaches were important to help us identify as many 3GCs as possible. The consensus sequence can be tuned to be more specific, given that the SSNs for aminotransferases demonstrate that they do segregate (Additional file 1: Fig. 3C) into subgroups of enzymes with more closely related aminotransferase sequences; the approach we employed here was aimed to be as broad as possible.

**Fig. 7:**
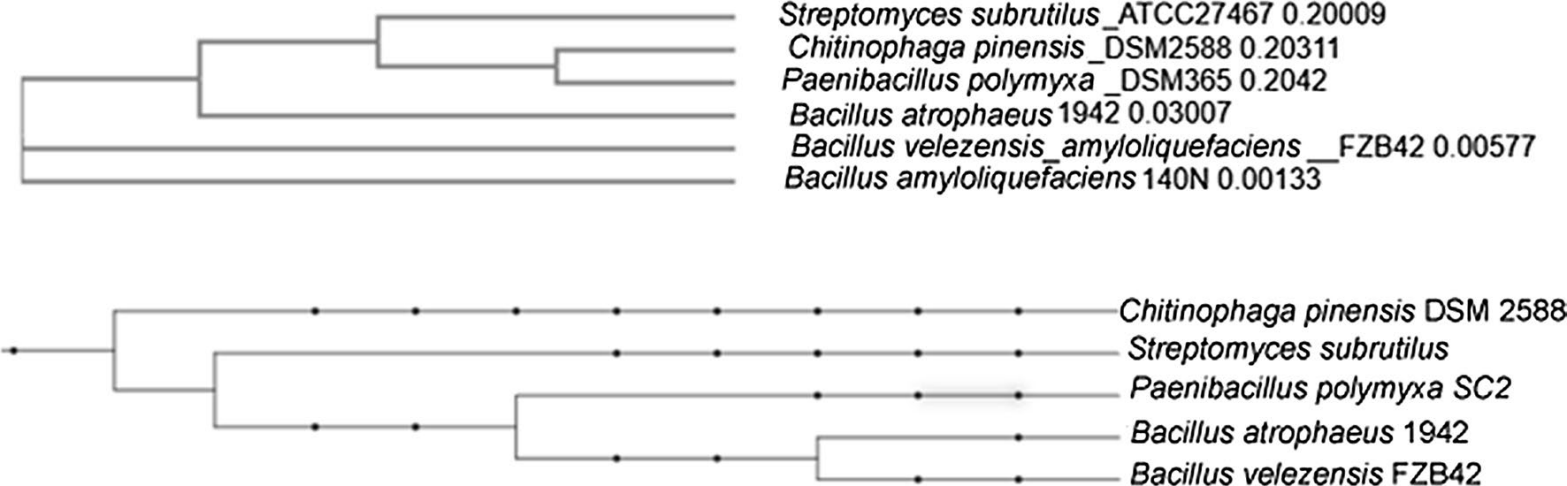
Simple unrooted neighbor-joining phylogenic tree without correction, using the aminotransferase of known azasugar producers. Tree created through Clustal-Omega (https://www.ebi.ac.uk/Tools/msa/clustalo/). B. Phylogenetic tree created through PhyloT (https://phylot.biobyte.de/) using NCBI taxonomy 16srRNA

Using the aminotransferase of known azasugar producers a phylogenetic network was generated with Clustal-Omega (McWilliam et al. 2013) for comparison with the taxonomic tree generated with PhyloT (https://phylot.biobyte.de/) for the same species using 16s RNA (Fig. 7). The relatedness in the taxonomic tree is not paralleled in the phylogenetic network for the aminotransferase sequences. The discrepancy between the trees perhaps represents the occurrence of a horizontal gene transfer event (Altenhoff et al. 2012). *C*. *pinensis* and *P. polymyxa* aminotransferase sequences appear to be more conserved with a common ancestor, potentially a horizontal gene transfer (HGT) event, and these non-Bacillus species are closer to *Bacillus atrophaeus*. While many species closely related to known azasugar producers lack a 3GC, very few have been tested for azasugar production. However, *B. subtilis* 168 has been identified as lacking a 3GC, and tested and failed to produce an azasugar under the same conditions as its closely related azasugar producing counterpart, *Bacillus velezensis* (Clark et al. 2011) lending some support for a HGT mechanism for the distribution of azasugar production capability in some organisms.

For a broader perspective we created a phylogenetic network using MEGA-X (Kumar et al. 2018) for all known and putative azasugar producers using the aminotransferase sequences (Additional file 1: Figure S8). Similar to the results for the known producers, we find that the same genera can be found in different regions of the network, indicating that the distribution of azasugar production is not strictly associated with a taxonomic distribution.

**Table 2:** Genera representing the bulk of putative azasugar producers. Hits for genera having less than 5 members omitted

Genus	Number of hits
*Paenibacillus*	135
*Bacillus*	126
*Xenorhabdus*	31
*Streptomyces*	39
*Photorhabdus*	25
*Chitinophaga*	14
*Clostridium*	8
*Pedobacter*	5
*Erwinia*	5
*Micromonospora*	5

Many of the putative producers we identified are commonly soil associated, such as *Bacillus* and *Paenibacillus*, however the breadth of niches for the putative producers we identified is vast and varied. They include marine sediment, soil throughout all seven continents, varying species of plants, food and even in human digestive flora. Of the putative azasugar producers, there are 55 strains of Xenorhabdus and Photorhabdus species (Table 2). These bacteria colonize the intestines of enteropathogenic nematodes and are released into an infected insect larva’s bloodstream (Goodrich‐Blair and Clarke 2007). A strain of Arsenophonus (Enterobacteriaceae), a symbiote to the agricultural pest, *Trialeurodes vaporariorum*, the greenhouse whitefly (Kapantaidaki et al. 2015) contains a putative 3GC. Another putative azasugar producer is *Bacillus rugosus* SPB7; this strain is symbiotic to the sea sponge *Spongia officinalis* (Bhattacharya et al. 2020). It is notable that a few strains of human pathogenic bacteria including *Streptococcus pneumonia* and *Cutibacterium acnes* have a three gene cluster (Additional file 1: Tables S1). Even though presence of a 3GC alone does not guarantee the expression of genes and ultimately production of azasugar, it does raise the question of why microbial species have the capacity to produce an azasugar. Future research could target these strains to investigate this question further. Azasugar biosynthesis is an understudied field thus far, despite the increased industrial interest in these molecules. The combination of SSNs and a new and unique consensus sequence allowed us to identify over 400 new putative azasugar producing microorganisms. Future studies will be aimed at exploring these species further in terms of their ability to produce an azasugar broadly, but also specifically allow for identification of the compound being produced, which in turn might allow us to begin to predict structural classes of azasugars based on sequence analyses. Finally, the species identified in this study encompass a wide range of niches, including those that might impact agriculture and human health.

## Supplementary Information

**Additional file 1.** Additional figures and Tables.

## Data Availability

A pdf file of all supplementary figures and tables mentioned in the article is available. The original xml files used to create SSN and GNN diagrams are available upon request, from the corresponding author.
