# Supplementary material for: Comparative genomic analysis of azasugar biosynthesis
Source: AMB Express. 2021 Aug 23;11:120. doi: 10.1186/s13568-021-01279-5 (PMC8382821; doi:10.1186/s13568-021-01279-5)
Supplement: Supplementary file 1 — Additional file 1. Additional figures and Tables. [file 13568_2021_1279_MOESM1_ESM.docx]

AMB Express

Comparative genomic analysis of azasugar biosynthesis

Beal, Hailey; Horenstein, Nicole
Department of Chemistry, University of Florida, Gainesville, FL 32611-7200, USA

[horen@chem.ufl.edu](mailto:horen@chem.ufl.edu), 01 352 392 9859

List of Contents:

**Figure S1**: SSN map of *B. velezensis* GutB1 (dehydrogenase) mapped with color from GabT1 (aminotransferase) 40%

**Figure S2**: SSN map of *B. velezensis* GutB1 (dehydrogenase) mapped with color from GabT1 (aminotransferase) 50%

**Figure S3**: A. *Bacillus velezensis* FZB42 GutB1 Percent Identity vs. Alignment Score. 40% identity correlates to Alignment Score threshold of 85 and 50% identity correlates to Alignment Score threshold of 95.B. *B. velezensis* GabT1 Percent Identity vs. Alignment Score. 40% identity correlates to Alignment Score of 110 and 50% Identity correlates to an Alignment score threshold of 140. For further analysis, Alignment score thresholds of 150, 155 and 180 were chosen to correlate with Percent Identities of 55, 60 and 70%.

**Figure S4**: *B. velezensis* FZB42 aminotransferase GabT1 SSN data with more stringent alignment scores. A. 55% Identity B.60% Identity C. 70% Identity. Known azasugar producers are assigned the following corresponding shapes: Parallelogram: *P. polmyxa* DSM365, Triangle*: C. pinensis* DSM2588, square: *B. velezensis* FZB42, diamond: *B. atrophaeus* 1942, and large circle*: B. amyloliquefaciens* 140N, Octagon: *S. subrutilis*

**Figure S5**: *B. velezensis* FZB42 dehydrogenase GutB1 SSN with increasingly stringent SSNs A. 55% Identity B. 60% Identity C. 70% identity. Known azasugar producers are identified as polygons according figure S4 above.

**Figure S6**: A. *C. pinensis* YktC1 SSN – Triangle: *C. pinensis* B. *B. velezensis* FZB42 YktC1 SSN – Square: *Bacillus velezensis,* Diamond*: B. atrophaeus*, Circle: *B. amyloliquefaciens* 140N. C. *C. pinensis* YktC1 GNN – only cluster 3 shows 3GC genome neighborhoods D. *B. velezensis* FZB42 YktC1 GNN – Clusters 1 and 20 show 3GC genome neighborhoods.

**Figure S7**: *P. polymyxa* DSM365 Phosphatase SSN

**Figure S8**: Unrooted Phylogenetic Tree of the known azasugar producers’ aminotransferase GabT1. Known producers are indicated with a black star next to the name.

**Figure S9:** Multiple Sequence Alignment of GabT1s from Azasugar Producers

**Figure S10**: Interpro analysis of GabT1 consensus sequence derived from putative azasugar producers found in this study. The gap of the PIRSF0000521 CD is the sequence AFRREPFPpqIxSfgLQVPD.

**Table S1:** Table of putative and known azasugar strains, both identified from SSNs and consensus sequence along with the gene names for the aminotransferase and dehydrogenase.


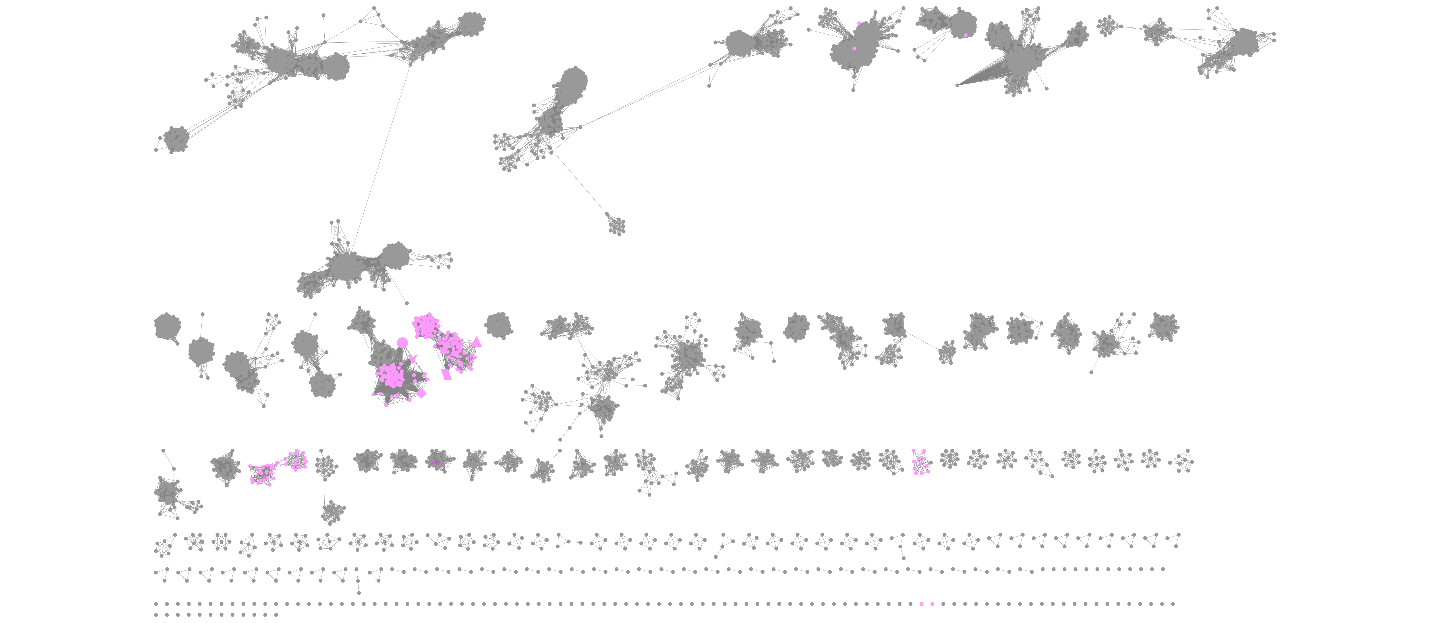


**Figure S1**


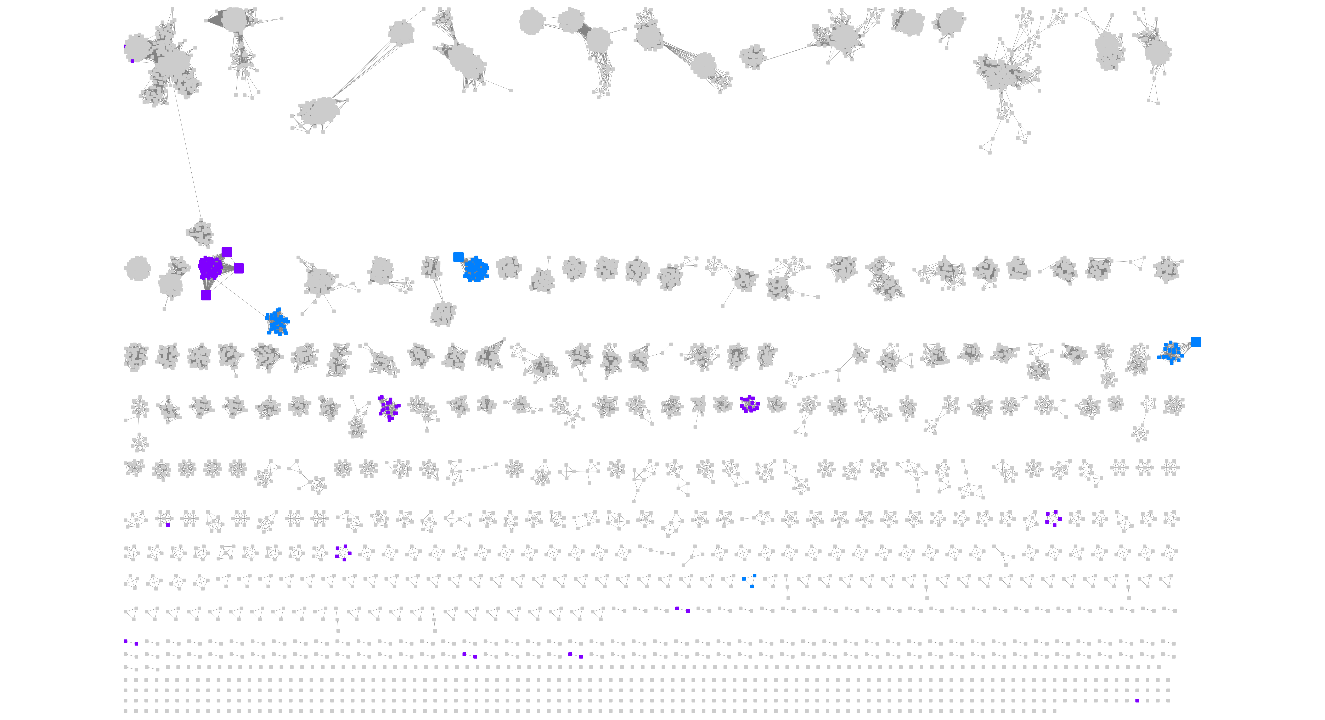


**Figure S2**


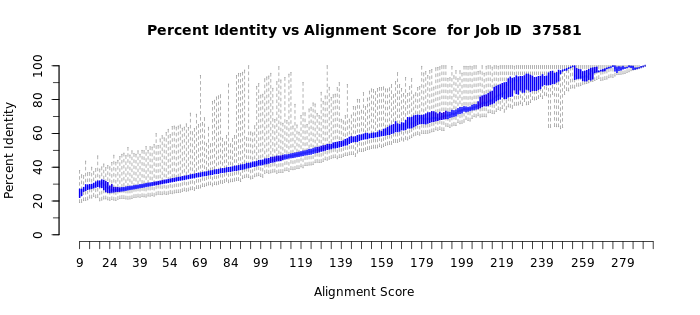


**A**

**B**


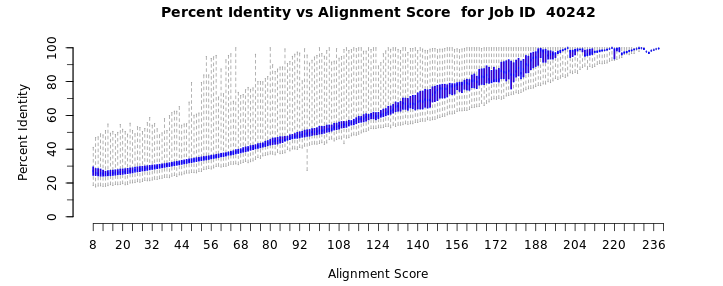


**Figure S3**


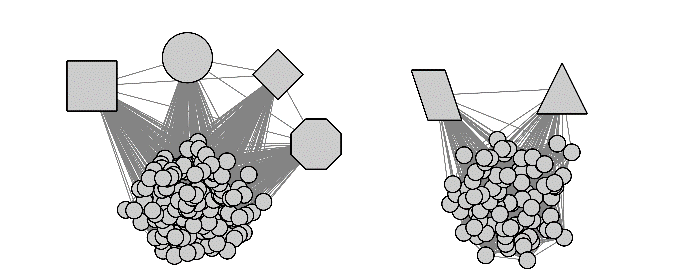

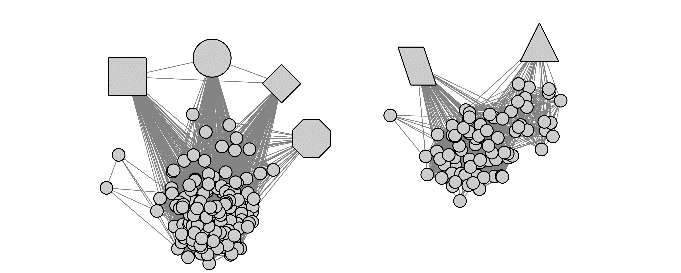

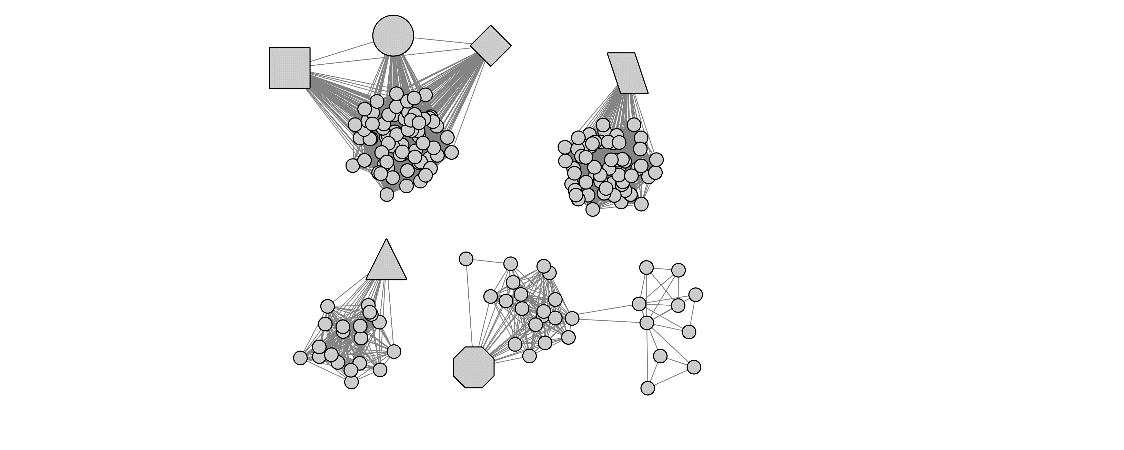


**C**

**B**

**A**

**Figure S4**


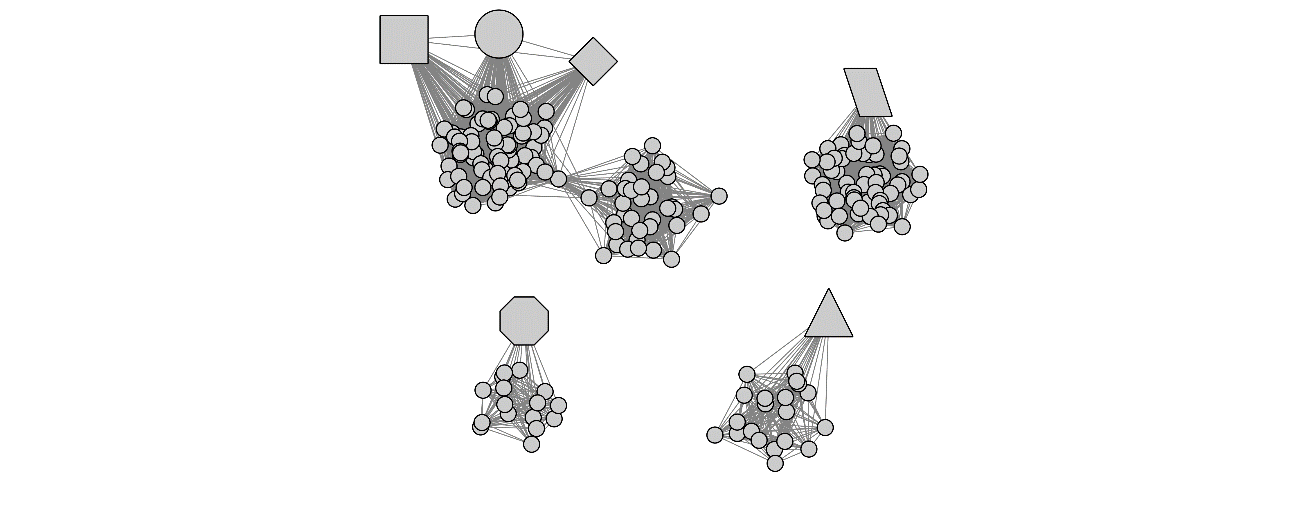

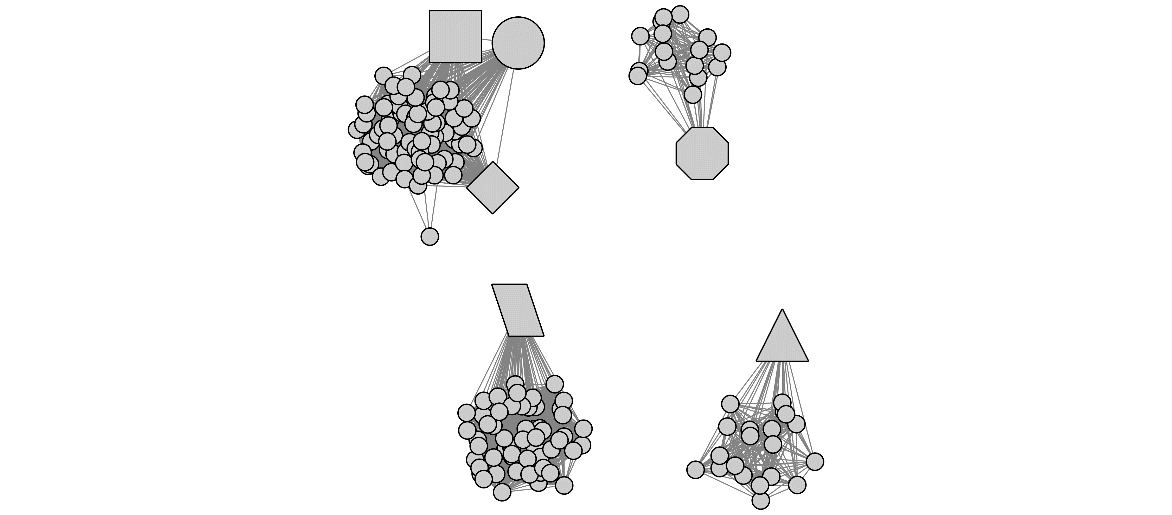

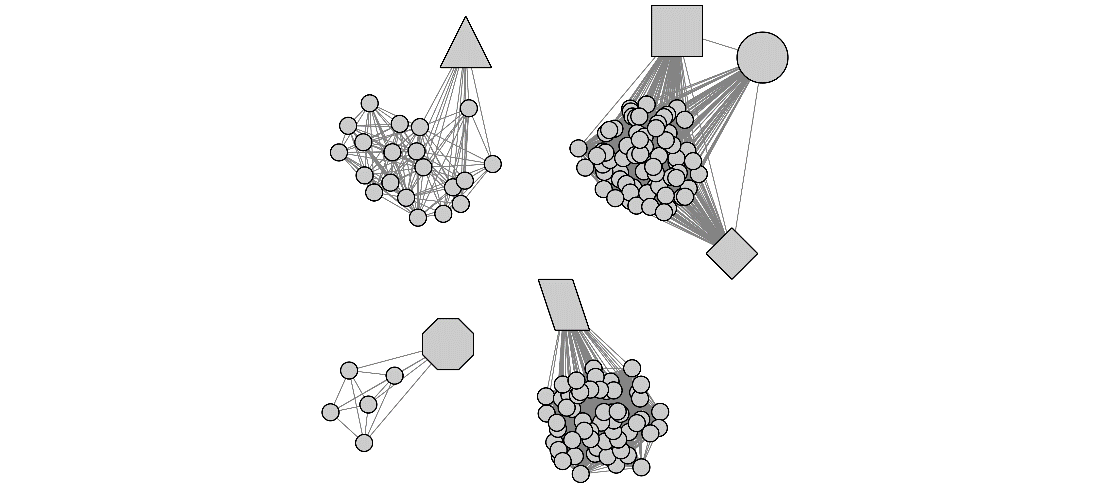


**A**

**C**

**B**

**Figure S5**


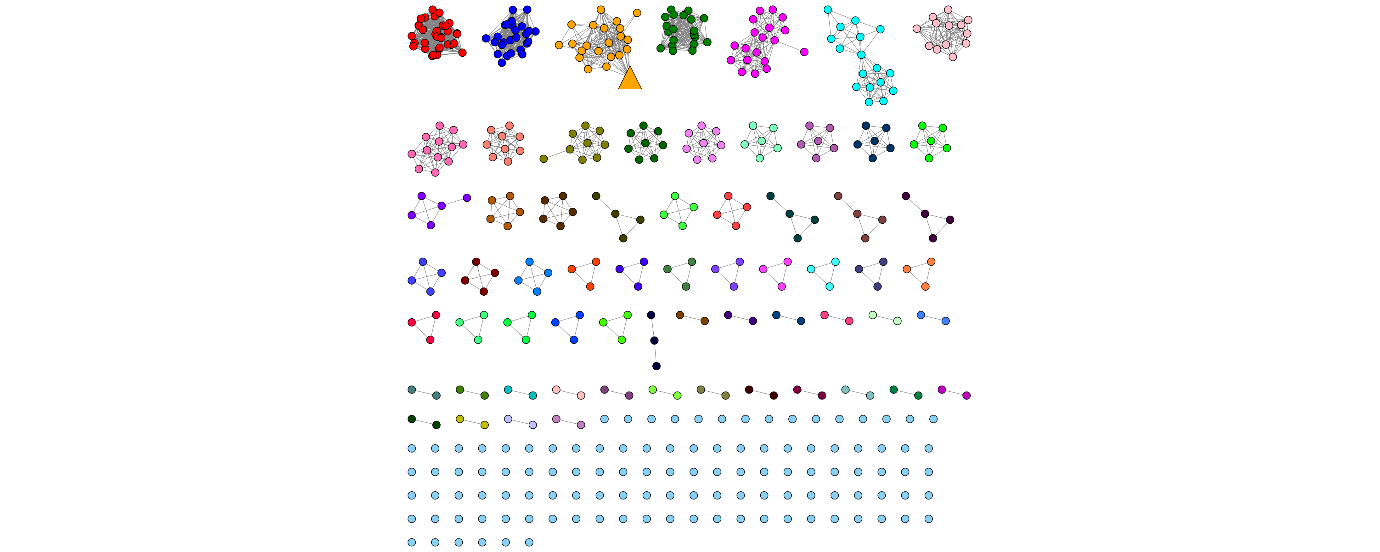

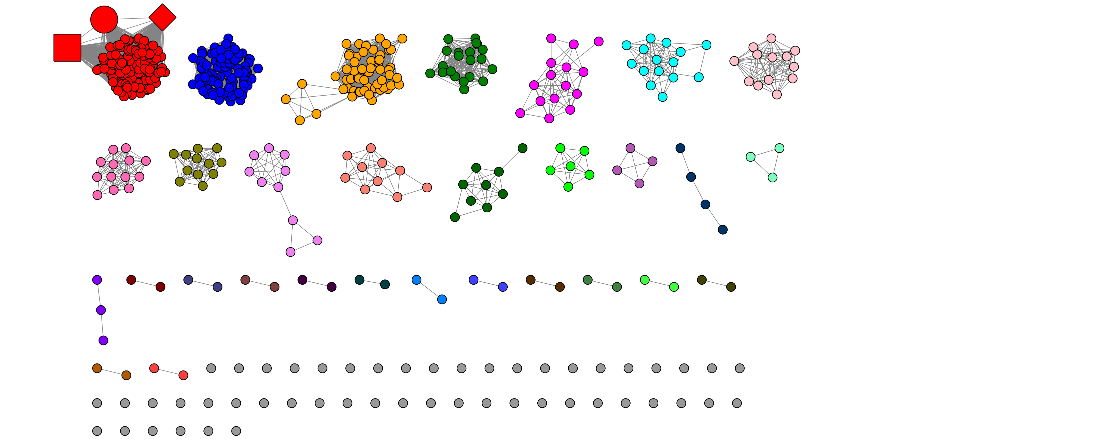


**B**

**A**


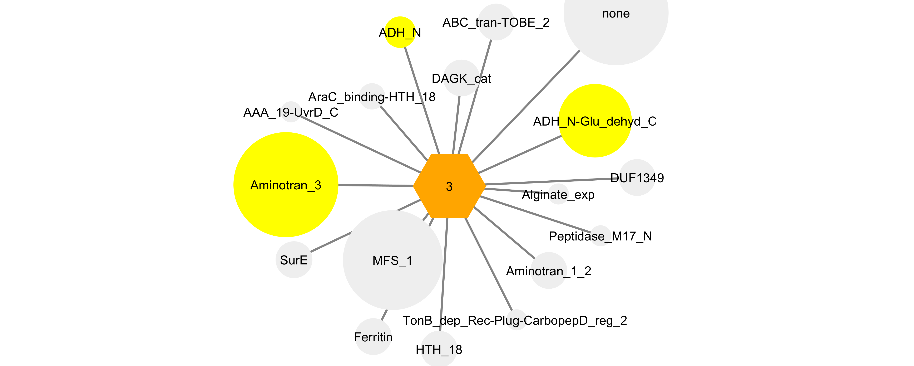

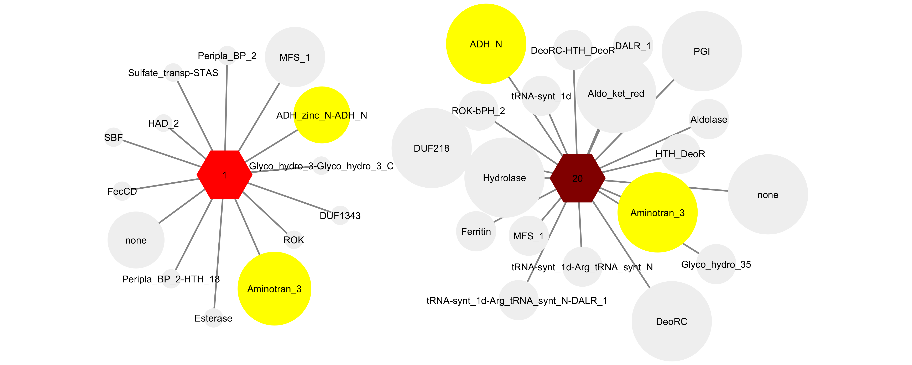


**D**

**C**

**Figure S6**


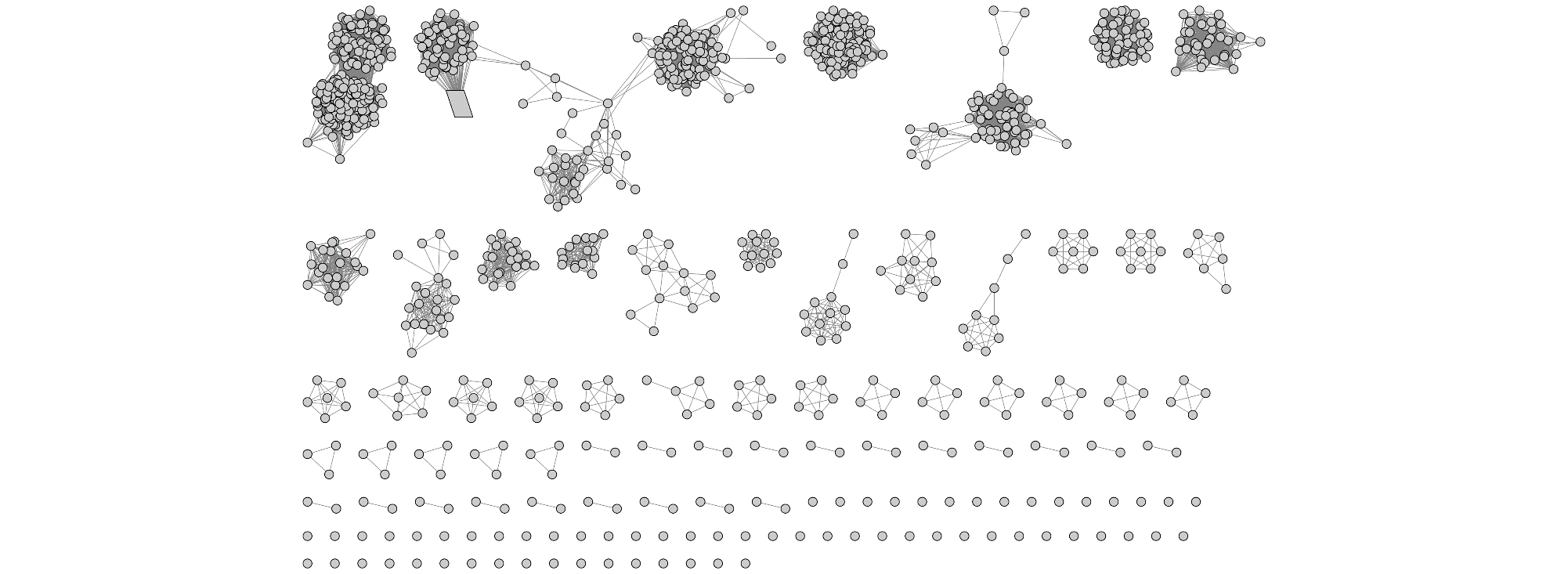


**Figure S7**


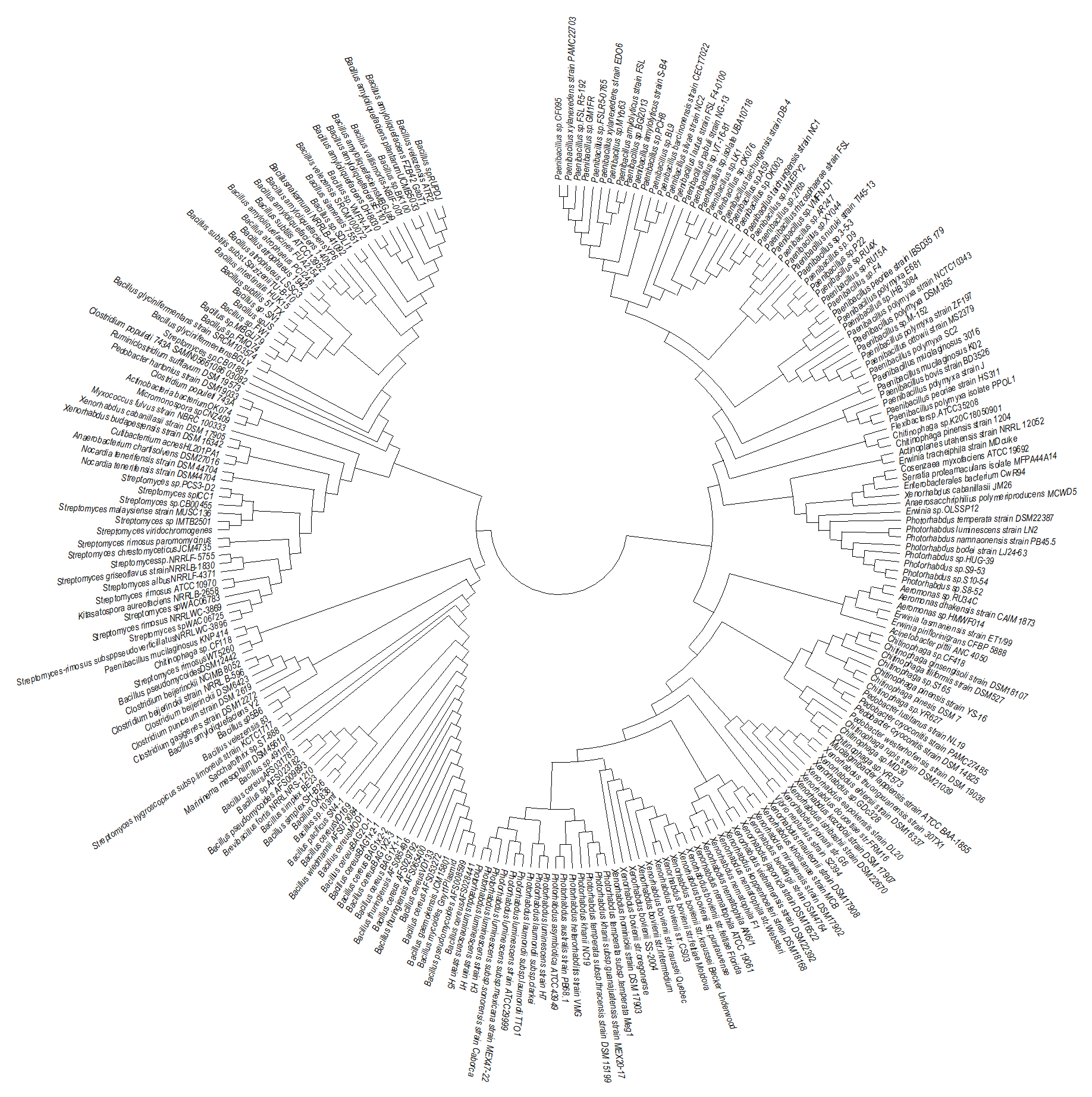


DNJ

DNJ

DAB-1

**Figure S8**


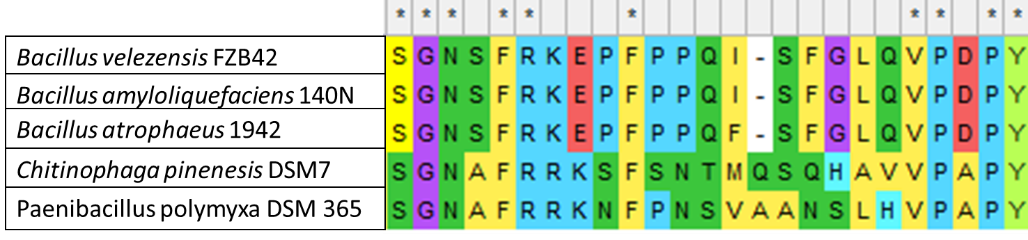


**Figure S9**


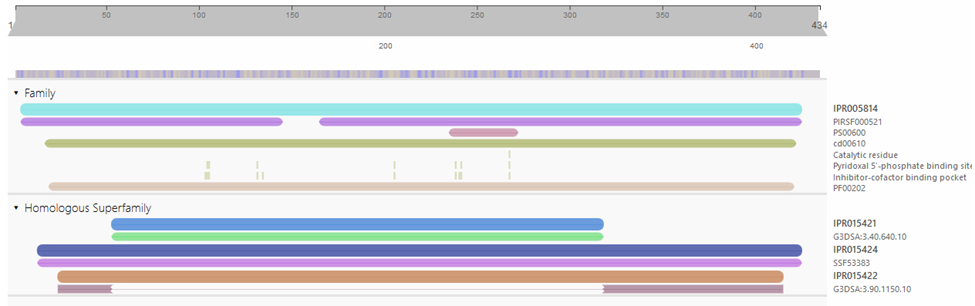


**Figure S10**

| **Name** | **Dehydrogenase** | **Aminotransferase** | **Identified by** | **Isolation Source** |
| --- | --- | --- | --- | --- |
| *Paenibacillus polymyxa* | A9Z39_18415 | A9Z39_18420 | CS | cow rumen |
| *Xenorhabdus khoisanae.* | AB204_06260 | AB204_06265 | SSN | nematode |
| *Bacillus thuringiensis* | ABB03_00165 | ABB03_00175 | CS | Soil |
| *Paenibacillus peoriae.* | ABE82_12340 | ABE82_12345 | SSN | Rhizobacterium |
| *Streptomyces viridochromogenes* | ACM01_26775 | ACM01_26765 | SSN/ CS | Soil |
| *Streptomyces rimosus subsp. rimosus.* | ADK43_15655 | ADK43_15665 | SSN | Soil |
| *Streptomyces rimosus subsp. pseudoverticillatus.* | ADK70_09850 | ADK70_09840 | SSN/ CS | Soil |
| *Streptomyces griseoflavus.* | ADK76_35430 | ADK76_35420 | SSN | Soil |
| *Streptomyces sp. NRRL WC-3701.* | ADK84_13425 | ADK84_13415 | SSN | - |
| *Streptomyces sp. NRRL F-5755.* | ADK86_12805 | ADK86_12815 | SSN/ CS | Soil |
| *Streptomyces albus subsp. albus.* | ADL21_15865 | ADL21_15855 | SSN/ CS | Soil |
| *Xenorhabdus sp. GDc328.* | AFK69_01055 | AFK69_01050 | SSN | nematode |
| *Bacillus velezensis TrigoCor1448* | AJ82_01160 | AJ82_01150 | CS | wheat plant |
| *Saccharothrix sp. ALI-22-I.* | ALI22I_04755 | ALI22I_04750 | SSN | rhizosphere soil – Utah Canyonlands National Park |
| *Paenibacillus polymyxa* | AM598_23530 | AM598_23525 | CS | cotton |
| *Paenibacillus xylanivorans.* | AMS66_11015 | AMS66_11020 | SSN/ CS | Decaying forest soil from Patagonia, Argentina |
| *Paenibacillus xylanivorans.* | AMS66_19400 | AMS66_19405 | SSN | Decaying forest soil from Patagonia, Argentina |
| *Bacillus amyloliquefaciens* | AN475_14440 | AN475_14430 | CS | - |
| *Paenibacillus polymyxa* | AOU00_24825 | AOU00_24830 | SSN/ CS | under epidermis tissue of stem: capsicum annuum(chili peppers) |
| *Paenibacillus bovis.* | AR543_10570 | AR543_10565 | SSN/ CS | raw yak milk |
| *Arsenophonus endosymbiont of Trialeurodes vaporariorum.* | ARTV_1927 | ARTV_1926 | SSN | - |
| *Paenibacillus sp. Leaf72.* | ASF12_04035 | ASF12_04030 | SSN | - |
| *Paenibacillus sp. IHB B 3084.* | ASL14_23995 | ASL14_24000 | SSN / CS | Soil |
| *Cutibacterium acnes* | Asn12ST33_03700 | Asn12ST33_03715 | CS | *Homo sapiens* |
| *Bacillus sp. SDLI1.* | AUL54_09755 | AUL54_09765 | SSN/ CS | larval gut of the stingless bee *Scaptotrigona depilis* (Paludo 16) |
| *Streptomyces sp. IMTB 2501.* | AV521_28485 | AV521_28475 | SSN/ CS | Soil |
| *Paenibacillus jamilae* | AV545_16505 | AV545_16500 | CS | Ulu Slim Hot Spring |
| *Paenibacillus elgii.* | AV654_30475 | AV654_30480 | SSN / CS | - |
| *Erwinia tracheiphila.* | AV903_26255 | AV903_26260 | SSN | *Cucumis sativus* |
| *Bacillus amyloliquefaciens* | AVM03_14780 | AVM03_14770 | CS | Korean traditional alcoholic beverage |
| *Bacillus velezensis* | AVR63_00025 | AVR63_00015 | CS | wheat anther |
| *Streptomyces sp. PCS3-D2.* | AW27_03610 | AW27_03600 | SSN/ CS | Soil |
| *Micromonospora rosaria.* | AWW66_07525 | AWW66_07520 | SSN | Soil |
| *Bacillus atrophaeus* | AXI57_18340 | AXI57_18350 | CS | Soil - Colorado |
| *Bacillus nakamurai.* | AXI58_13925 | AXI58_13935 | SSN | Soil |
| *Bacillus nakamurai* | AXI59_18440 | AXI59_18450 | CS | Soil - Argentina |
| *Pedobacter cryoconitis.* | AY601_1441 | AY601_1440 | SSN | Soil |
| *Paenibacillus polymyxa* | AZE31_08945 | AZE31_08940 | CS | worm compost |
| *Paenibacillus sp. VT-16-81.* | B2I21_21725 | B2I21_21720 | SSN/ CS | Human Saliva |
| *Paenibacillus mucilaginosus K02.* | B2K_11180 | B2K_11175 | SSN | Soil |
| *Bacillus velezensis* | B6257_10905 | B6257_10895 | CS | gut |
| *Bacillus mycoides strain Gnyt plasmid unnamed3* | B7492_32520 | B7492_32525 | SSN | - |
| *Bacillus mycoides.* | B7492_32530 | B7492_32520 | SSN | Soil |
| *Bacillus vallismortis.* | B9C48_01020 | B9C48_01010 | SSN | Soil - China |
| *Photorhabdus luminescens BA1.* | BA1DRAFT_03297 | BA1DRAFT_03296 | SSN | *Heterorhabditis bacteriophora* |
| *Bacillus gaemokensis.* | BAGA_22055 | BAGA_22065 | SSN/ CS | tidal flat sediments of the Yellow Sea in the region of Tae-An (Jung 2016) |
| *Bacillus amyloliquefaciens TA208* | BAMTA208_00810 | BAMTA208_00800 | CS | - |
| *Bacillus amyloliquefaciens* | BAMY_01015 | BAMY_01005 | CS | rhizosphere of peanut |
| *Bacillus amyloliquefaciens* | BARD7_00201 | BARD7_00199 | CS | fermented soybean paste |
| *Bacillus atrophaeus (strain 1942)* | BATR1942_19425 | BATR1942_19415 | SSN/ CS | Rhizosphere/soil |
| *Bacillus subtilis* | BAX60_06670 | BAX60_06660 | CS | crude oil contaminated soil |
| *Paenibacillus sp. BIHB4019.* | BBD42_19500 | BBD42_19505 | SSN | - |
| *Paenibacillus ferrarius.* | BC351_18650 | BC351_18655 | SSN | - |
| *Actinoplanes xinjiangensis.* | BC793_13760 | BC793_13761 | SSN | - |
| *Bacillus velezensis* | BCBMB205_01600 | BCBMB205_01580 | CS | rhizosphere soil of field-grown rice |
| *Bacillus thuringiensis* | BCM43_28085 | BCM43_28075 | CS | Ginseng |
| *Bacillus subtilis* | BCV50_11830 | BCV50_11840 | CS | elk droppings |
| *Paenibacillus sp. SSG-1* | BCV73_19845 | BCV73_19830 | CS | soil - China |
| *Xenorhabdus cabanillasii.* | BDD26_1528 | BDD26_1527 | SSN | Soil - Texas |
| *Bacillus subtilis* | BG616_00545 | BG616_00535 | CS | organic manure |
| *Bacillus glycinifermentans.* | BGLY_0303 | BGLY_0305 | SSN | Soybean paste |
| *Bacillus cereus.* | BHL35_00605 | BHL35_00615 | SSN | sesame seed |
| *Paenibacillus sp. RU15A.* | BJV48_1825 | BJV48_1826 | SSN | - |
| *Paenibacillus pabuli* | BK122_11240 | BK122_11245 | CS | milk |
| *Paenibacillus lautus* | BK123_17235 | BK123_17240 | SSN/ CS | milk |
| *Paenibacillus amylolyticus* | BK124_01425 | BK124_01430 | CS | milk |
| *Paenibacillus amylolyticus* | BK129_15925 | BK129_15930 | CS | milk |
| *Paenibacillus amylolyticus.* | BK131_07580 | BK131_07585 | SSN | - |
| *Paenibacillus peoriae* | BK135_25280 | BK135_25285 | CS | milk |
| *Paenibacillus amylolyticus.* | BK136_00495 | BK136_00490 | SSN | milk |
| *Paenibacillus amylolyticus.* | BK136_05990 | BK136_05995 | SSN/ CS | milk |
| *Paenibacillus rhizosphaerae.* | BK138_11155 | BK138_11160 | SSN/ CS | milk |
| *Paenibacillus sp. FSL R5-0765.* | BK141_08180 | BK141_08185 | SSN | milk |
| *Paenibacillus sp. FSL R5-0765.* | BK141_14035 | BK141_14030 | SSN | milk |
| *Paenibacillus peoriae* | BK143_25390 | BK143_25395 | CS | milk |
| *Erwinia sp. OLSSP12.* | BK416_13460 | BK416_13455 | SSN | Whole insect macerate of *Oris Laevigatus*(flower bug) |
| *Bacillus amyloliquefaciens* | BKP66_17175 | BKP66_17185 | CS | - |
| *Frankia sp. BMG5.30.* | BL254_21990 | BL254_22005 | SSN | root nodule |
| *Bacillus sp. FMQ74.* | BLL41_17105 | BLL41_17115 | SSN/ CS | raw milk |
| *Bacillus velezensis.* | BMJ37_01140 | BMJ37_01150 | SSN | soil |
| *Bacillus cereus* | BN2127_JRS1_06570 | BN2127_JRS1_06568 | CS | - |
| *Paenibacillus sp. P22.* | BN871_BV_00180 | BN871_BV_00190 | SSN | - |
| *Bacillus pseudomycoides DSM 12442.* | bpmyx0001_52510 | bpmyx0001_52530 | SSN/ CS | - |
| *Paenibacillus xylanexedens.* | BS614_17830 | BS614_17835 | SSN | sediment |
| *Paenibacillus xylanexedens.* | BS614_21625 | BS614_21620 | SSN/ CS | sediment |
| *Bacillus inaquosorum KCTC 13429* | BSI_38140 | BSI_38160 | CS | inaquosorum |
| *Bacillus mycoides* | BTJ45_03501 | BTJ45_03503 | CS | - |
| *[Flexibacter] sp. ATCC 35208.* | BW716_26160 | BW716_26155 | SSN/ CS | Grass |
| *Streptomyces sp. 2333.5.* | BX264_0990 | BX264_0988 | SSN | - |
| *Paenibacillus sp. lzh-N1* | C0638_20900 | C0638_20895 | CS | Soil- China |
| *Paenibacillus sp. PCH8.* | C0Q44_19565 | C0Q44_19560 | SSN/ CS | High altitude soil - India |
| *Paenibacillus sp. PCH8.* | C0Q44_23185 | C0Q44_23180 | SSN | High altitude soil - India |
| *Paenibacillus sp. FSL R5-192.* | C161_14123 | C161_14118 | SSN | Pasteurized milk |
| *Paenibacillus sp. FSL R5-192.* | C161_22079 | C161_22074 | SSN/ CS | Pasteurized milk |
| *Paenibacillus sp. FSL H7-689* | C170_20085 | C170_20090 | CS | pasteurized milk |
| *Paenibacillus polymyxa* | C1A50_2498 | C1A50_2499 | CS | the tomato root in Nanchang |
| *Paenibacillus polymyxa* | C1I59_11405 | C1I59_11410 | CS | rhizosphere |
| *Paenibacillus terrae* | C1I60_18605 | C1I60_18610 | CS | rhizosphere |
| *Paenibacillus polymyxa* | C1T20_10860 | C1T20_10855 | CS | - |
| *Paenibacillus sp. F4.* | C1T21_12555 | C1T21_12560 | SSN/ CS | - |
| *Bacillus cereus* | C1T25_01395 | C1T25_01385 | CS | Jackal Excreta |
| *Bacillus sp. MBGLi79.* | C1T29_01275 | C1T29_01265 | SSN | Lion Excreta |
| *Bacillus sp. MBGLi97.* | C1T30_26680 | C1T30_26690 | SSN | Lion Excreta |
| *Bacillus velezensis.* | C3Z10_01025 | C3Z10_01015 | SSN | - |
| *Paenibacillus sp. ICGEB2008* | C400_09890 | C400_09895 | CS |  |
| *Photorhabdus khanii subsp. guanajuatensis.* | C5467_15925 | C5467_15920 | SSN | *Heterorhabditis bacteriophora* |
| *Photorhabdus luminescens subsp. mexicana.* | C5468_22275 | C5468_22280 | SSN | *Heterorhabditis bacteriophora mexicana* |
| *Paenibacillus peoriae.* | C5G87_01575 | C5G87_01570 | SSN/ CS | - |
| *Bacillus sp. ZY-1-1* | C5I45_18560 | C5I45_18570 | CS | gut content *Holotrichia parallela* |
| *Bacillus atrophaeus* | C6371_18290 | C6371_18300 | CS | soil from salt lake |
| *Streptomyces sp. P3.* | C6376_24540 | C6376_24535 | SSN | - |
| *Photorhabdus luminescens* | C6H65_13205 | C6H65_13200 | SSN | Soil: Heterorhabditis |
| *Photorhabdus luminescens* | C6H66_04390 | C6H66_04385 | SSN | Soil: Heterorhabditis |
| *Photorhabdus luminescens* | C6H68_18750 | C6H68_18755 | SSN | Soil: Heterorhabditis |
| *Photorhabdus luminescens* | C6H69_19770 | C6H69_19765 | SSN | Heterorhabditis |
| *Bacillus atrophaeus* | C6W23_12515 | C6W23_12505 | CS | Soil- China |
| *Chitinophaga sp. S165.* | C7475_105130 | C7475_105131 | SSN | Soil/plant associated |
| *Bacillus sp. VMFN-A1.* | C7819_1055 | C7819_1053 | SSN/ CS | Seeds of *Opuntia robusta* |
| *Paenibacillus sp. VMFN-D1.* | C7820_0086 | C7820_0087 | SSN | Mexico: El Freno |
| *Brevibacillus fortis.* | C7R93_11895 | C7R93_11885 | SSN/ CS | soil |
| *Paenibacillus elgii.* | C8Z91_14080 | C8Z91_14085 | SSN/ CS | - |
| *Paenibacillus taichungensis* | CA599_09460 | CA599_09455 | CS | Low level radioactive waste repository |
| *Clostridium beijerinckii (strain ATCC 51743 / NCIMB 8052)* | Cbei_3890 | Cbei_3891 | SSN | - |
| *Bacillus intestinalis.* | CE489_19370 | CE489_19380 | SSN | - |
| *Bacillus velezensis* | CEG11_01020 | CEG11_01010 | CS | Root endosphere |
| *Paenibacillus sp. 7523-1.* | CHH60_05460 | CHH60_05465 | SSN | - |
| *Bacillus siamensis.* | CHH79_15490 | CHH79_15500 | SSN | Uncut heroin sample |
| *Paenibacillus sp. 7516.* | CHI14_05300 | CHI14_05305 | SSN | - |
| *Clostridium beijerinckii* | CIBE_4689 | CIBE_4690 | SSN | soil |
| *Paenibacillus sp. RUD330* | CIC07_04160 | CIC07_04155 | CS | *Euglena gracilis* from city ponds |
| *Enterobacterales bacterium* | CIG19_09630 | CIG19_09625 | SSN | *Fragaria ananassa* (strawberry) leaf |
| *Bacillus velezensis* | CJ467_08240 | CJ467_08250 | CS | Garden soil |
| *Bacillus subtilis* | CJ481_03880 | CJ481_03890 | CS | Plant root |
| *Paenibacillus sp. XY044.* | CJP46_32635 | CJP46_32640 | SSN/ CS | Stems of Maoxie tea plant(C*amellia sinesis* cv. Maoxie) |
| *Chitinophaga sp. MD30.* | CK934_01200 | CK934_01205 | SSN | Air conditioner condensate pipe: Hawaii |
| *Photorhabdus laumondii subsp. clarkei.* | CKY01_19580 | CKY01_19585 | SSN | soil |
| *Photorhabdus bodei.* | CKY02_19785 | CKY02_19790 | SSN | soil |
| *Photorhabdus sp. S9-53.* | CKY03_19615 | CKY03_19620 | SSN | soil |
| *Photorhabdus sp. S8-52.* | CKY04_19115 | CKY04_19120 | SSN | soil |
| *Photorhabdus sp. S10-54.* | CKY05_19595 | CKY05_19600 | SSN | soil |
| *Photorhabdus sp. HUG-39.* | CKY10_20580 | CKY10_20575 | SSN | soil |
| *Clostridium beijerinckii* | CLOBE_39730 | CLOBE_39720 | SSN | soil |
| *Clostridium puniceum.* | CLPUN_27590 | CLPUN_27600 | SSN | Potato (Root Rhizome) |
| *Micromonospora sp. CNZ309.* | CLT72_0890 | CLT72_0888 | SSN | Marine sediment Pacific Ocean(San Diego) |
| *Chitinophaga ginsengisoli.* | CLV42_12311 | CLV42_12310 | SSN | - |
| *Umezawaea tangerine* | CLV43_104683 | CLV43_104684 | SSN | Soil - Japan |
| *Bacillus stercoris* | CM50_20850 | CM50_20840 | CS | food-waste degradation bioreactor - Austraila |
| *Bacillus sp. AFS023182.* | CN288_27305 | CN288_27295 | SSN/ CS | Soybean Leaf |
| *Bacillus thuringiensis* | CN325_18880 | CN325_18890 | CS | soybean core |
| *Bacillus cereus.* | CN354_20970 | CN354_20960 | SSN | Core of Corn |
| *Bacillus pseudomycoides.* | CN613_30185 | CN613_30175 | SSN | Corn Root |
| *Bacillus wiedmannii.* | CN626_14630 | CN626_14620 | SSN | Soil |
| *Bacillus pseudomycoides.* | CN641_26195 | CN641_26185 | SSN | soil |
| *Bacillus cereus.* | CN957_30760 | CN957_30770 | SSN/ CS | soil |
| *Bacillus cereus* | COC47_20010 | COC47_20000 | CS | Corn Core |
| *Bacillus cereus.* | COE15_27440 | COE15_27450 | SSN | Soybean Leaf |
| *Bacillus cereus.* | COI69_29030 | COI69_29020 | SSN | soil |
| *Bacillus cereus* | COI97_23760 | COI97_23770 | CS | plant core |
| *Bacillus thuringiensis.* | COJ15_17830 | COJ15_17820 | SSN | Plant Core |
| *Bacillus cereus.* | COK05_08860 | COK05_08850 | SSN | - |
| *Bacillus thuringiensis.* | COK72_32320 | COK72_32310 | SSN/ CS | soil |
| *Bacillus pseudomycoides* | CON64_09630 | CON64_09620 | CS | Corn leaf |
| *Bacillus pseudomycoides* | CON79_25065 | CON79_25055 | CS | corn core |
| *Streptomyces subrutilus.* | CP968_06020 | CP968_06030 | SSN/ CS | soil |
| *Streptomyces albofaciens JCM 4342* | CP973_29355 | CP973_29345 | CS | soil |
| *Streptomyces nodosus.* | CP978_00050 | CP978_00055 | SSN | - |
| *Streptomyces rimosus.* | CP984_08855 | CP984_08845 | SSN | - |
| *Chitinophaga pinensis (strain ATCC 43595 / DSM 2588 / NCIB 11800 / UQM2034)* | Cpin_2153 | Cpin_2154 | SSN | Pine Litter in Australia – Known producer of DAB-1 |
| *Paenibacillus sp. AR247.* | CPT76_21350 | CPT76_21355 | SSN/ CS | Liquor from sugarcane’s bagasse |
| *Paenibacillus sp. MYb63.* | CQ043_07845 | CQ043_07840 | SSN | Soil: Heterorhabditis |
| *Paenibacillus sp. MYb63.* | CQ043_12840 | CQ043_12835 | SSN | Rotting apple – *Caenorhabiditis elegans* MY316 |
| *Paenibacillus sp. LK1.* | CS562_06195 | CS562_06200 | SSN | Rhizosphere of *Auricularia auricular* (fungus) |
| *Paenibacillus sp. LK1.* | CS562_22885 | CS562_22880 | SSN | Rhizosphere of *Auricularia auricular* (fungus) |
| *Bacillus sp. SN1.* | CVV77_14275 | CVV77_14265 | SSN | *Stegodyphus dumicola* (African social spider) |
| *Bacillus siamensis.* | CWD84_20955 | CWD84_20965 | SSN | - |
| *Aeromonas dhakensis.* | CX648_13425 | CX648_13420 | SSN | *Oreochromis niloticus* (tilapia) |
| *Paenibacillus sp. BGI2013.* | CXK86_10125 | CXK86_10130 | SSN | the small intestine of a 50000 y.o. permafrost *Bison priscus* mummy |
| *Paenibacillus sp. BGI2013.* | CXK86_15715 | CXK86_15710 | SSN | the small intestine of a 50000 y.o. permafrost *Bison priscus* mummy |
| *Bacillus atrophaeus UCMB-5137* | D068_cds01490 | D068_cds01470 | CS | rhizosphere |
| *Bacillus velezensis* | D0872_14710 | D0872_14720 | CS | Sorghum |
| *Bacillus amyloliquefaciens* | D2M30_0202 | D2M30_0200 | SSN | Rhizosphere of *Lolium perenne*(Rye Grass) on a rock phosphorous mine China |
| *Streptomyces hoynatensis.* | D7294_14200 | D7294_14205 | SSN | - |
| *Aeromonas sp. HMWF014.* | DBR19_05200 | DBR19_05195 | SSN | Water Michigan |
| *Trueperella pyogenes.* | DBV13_10190 | DBV13_10185 | SSN | - |
| *Paenibacillus taichungensis.* | DC345_04450 | DC345_04455 | SSN | soil |
| *Paenibacillus taichungensis.* | DC345_22165 | DC345_22170 | SSN | soil |
| *Streptomyces venezuelae.* | DEJ50_03080 | DEJ50_03075 | SSN | - |
| *Paenibacillus pabuli* | DET54_114150 | DET54_114149 | CS | Soil - Pennsylvania |
| *Paenibacillus pabuli.* | DET54_117136 | DET54_117137 | SSN | Soil -Pennsylvania |
| *Paenibacillus pabuli* | DET56_102106 | DET56_102105 | CS | Soil - Pennsylvania |
| *Bacillus amyloliquefaciens* | DEU43_1075 | DEU43_1073 | SSN/ CS | Rice endophyte |
| *Paenibacillus taichungensis.* | DEU73_102106 | DEU73_102105 | SSN | - |
| *Paenibacillus taichungensis.* | DEU73_11711 | DEU73_11710 | SSN | - |
| *Bacillus subtilis.* | DFO69_3709 | DFO69_3711 | SSN/ CS | - |
| *Paenibacillus barcinonensis.* | DFQ00_104303 | DFQ00_104302 | SSN | - |
| *Paenibacillus barcinonensis.* | DFQ00_12916 | DFQ00_12917 | SSN/ CS | - |
| *Anaerobacterium chartisolvens.* | DFR58_13917 | DFR58_13919 | SSN/ CS | - |
| *Nocardia tenerifensis.* | DFR70_112247 | DFR70_112248 | SSN | - |
| *Bacillus pseudomycoides* | DJ92_5559 | DJ92_5557 | CS | - |
| *Streptomyces sp. WAC 06725.* | DMH15_13055 | DMH15_13065 | SSN/ CS | soil - Nigeria |
| *Streptomyces sp. WAC 06783.* | DMH18_24935 | DMH18_24945 | SSN/ CS | Soil- France |
| *Bacillus sp. JAS24-2.* | DN407_29680 | DN407_29690 | SSN | Soil- Poland |
| *Paenibacillus silvae.* | DN757_05105 | DN757_05110 | SSN/ CS | Gold-Copper mine China |
| *Paenibacillus silvae.* | DN757_15960 | DN757_15955 | SSN | Gold-Copper mine China |
| *Streptomyces sp. ICC4* | DRB89_29580 | DRB89_29570 | CS | ron Curtain Cave |
| *Streptomyces sp. ICC1.* | DRB96_30095 | DRB96_30085 | SSN | Canadian Cave |
| *Anaerosacchriphilus polymeriproducens.* | DWV06_12820 | DWV06_12830 | SSN | - |
| *Bacillus atrophaeus* | DX926_12400 | DX926_12390 | CS | Soil - Mexico |
| *Chitinophaga sp. K20C18050901.* | DXN04_28335 | DXN04_28330 | SSN/ CS | Forest soil china |
| *Micromonospora fluostatini.* | E1091_05130 | E1091_05135 | SSN | soybean core |
| *Micromonospora sp. KC723.* | E1165_00225 | E1165_00220 | SSN | Core of Corn |
| *Paenibacillus amylolyticus.* | E2R58_10800 | E2R58_10795 | SSN/ CS | Corn Root |
| *Bacillus velezensis* | E4T61_01025 | E4T61_01015 | CS | Soi- Brazil |
| *Bacillus velezensis* | EBA29_00182 | EBA29_00180 | CS | Mango Ochard |
| *Paenibacillus amylolyticus* | EC604_19545 | EC604_19540 | CS | - |
| *Photorhabdus temperata.* | EDD23_4991 | EDD23_4992 | SSN | Soil |
| *Paenibacillus xylanexedens.* | EDO6_01455 | EDO6_01454 | SSN | soil |
| *Paenibacillus xylanexedens.* | EDO6_04264 | EDO6_04265 | SSN | soil |
| *Paenibacillus sp. M-152.* | EGM68_12280 | EGM68_12285 | SSN/ CS | Mud sample India |
| *Peribacillus simplex.* | EI200_11565 | EI200_11575 | SSN/ CS | Rhizophere of Corn |
| *Streptomyces ficellus.* | EIZ62_01070 | EIZ62_01075 | SSN | - |
| *Streptomyces ficellus.* | EIZ62_06800 | EIZ62_06810 | SSN/ CS | - |
| *Paenibacillus sp. 3-5-3.* | EJP77_01485 | EJP77_01490 | SSN | Plant Core |
| *Paenibacillus polymyxa* | EL23_24235 | EL23_24240 | SSN | - |
| *Paenibacillus sp. VT-400* | EL84_24460 | EL84_24455 | CS | human saliva |
| *Photorhabdus luminescens subsp. sonorensis.* | EP164_18340 | EP164_18345 | SSN | *Heterorhaditis sonorensis* |
| *Erwinia piriflorinigrans CFBP 5888.* | EPIR_3205 | EPIR_3206 | SSN | - |
| *Streptococcus pneumoniae* | ERS020178_03265 | ERS020178_03267 | CS | nasopharynx |
| *Streptomyces sioyaensis.* | EST54_03600 | EST54_03590 | SSN | corn core |
| *Streptomyces sp. TM32.* | EST92_00930 | EST92_00940 | SSN | soil |
| *Paenibacillus tyrfis* | ET33_20795 | ET33_20790 | CS | Tropical peat swamp soil |
| *Erwinia tasmaniensis (strain DSM 17950 / CIP 109463 / Et1/99)* | ETA_29000 | ETA_29010 | SSN | - |
| *Bacillus velezensis.* | ETK69_01410 | ETK69_01400 | SSN |  |
| *Bacillus sp. BK100.* | EV570_1085 | EV570_1083 | SSN | - |
| *Acinetobacter pittii ANC 4050.* | F931_01507 | F931_01506 | SSN | - |
| *Streptomyces piniterrae* | FCH28_08210 | FCH28_08200 | CS | Rhizosphere of Pinusyunnanensis |
| *Paenibacillus sp. UASWS1643* | FE296_24655 | FE296_24660 | CS | tomato stem |
| *Chitinophaga pinensis.* | FEF09_18915 | FEF09_18920 | SSN | Rotten soil India |
| *Actinopolyspora biskrensis* | FHR84_RS02825 | FHR84_000572 | CS | - |
| *Chitinophaga pinensis.* | FHW35_10942 | FHW35_10941 | SSN | soil - Nebraska |
| *Paenibacillus sp. 597.* | FHW43_102961 | FHW43_102962 | SSN |  |
| *Actinoplanes teichomyceticus.* | FHX34_10593 | FHX34_10594 | SSN | Soil India |
| *Bacillus pacificus.* | FHY68_21100 | FHY68_21110 | SSN/ CS | *Ostrinia nubilalis* (corn borer) Vietnam |
| *Myxococcus sp. AM401.* | FJV41_08160 | FJV41_08155 | SSN | Soil UK |
| *Bacillus glycinifermentans* | FKN04_02835 | FKN04_02825 | CS | Soil - Pakistan |
| *Paenibacillus ottowii.* | FKV70_23835 | FKV70_23840 | SSN | Pasteurized solution sample from bovine manure |
| *Paenibacillus polymyxa* | FQU75_03795 | FQU75_03800 | SSN/CS | Soil China |
| *Candidatus Frankia datiscae.* | FsymDg_2613 | FsymDg_2611 | SSN | the small intestine of a 50000 y.o. permafrost *Bison priscus* mummy |
| *Paenibacillus elgii* | G3T11_08495 | G3T11_08500 | CS | roots |
| *Bacillus sp. LUNF1.* | G4O42_01020 | G4O42_01010 | SSN | - |
| *Bacillus rugosus* | G8D97_00795 | G8D97_00785 | CS | *Spongia officinalis* |
| *Bacillus sp. EKM420B* | G9F48_18985 | G9F48_18975 | CS | watermelon |
| *Bacillus sp. EKM208B* | G9F75_15370 | G9F75_15380 | CS | cantaloupe seeds |
| *Paenibacillus sp. EKM206P* | G9G53_23240 | G9G53_23245 | CS | cantaloupe seeds |
| *Paenibacillus sp. EKM212P* | G9G54_23680 | G9G54_23675 | CS | cantaloupe seeds |
| *Paenibacillus sp. EKM211P* | G9G57_15340 | G9G57_15335 | CS | cantaloupe seeds |
| *Paenibacillus sp. EKM202P* | G9G63_23120 | G9G63_23115 | CS | cantaloupe seeds |
| *Paenibacillus silvae* | GCM10008014_01060 | GCM10008014_01050 | CS | - |
| *Paenibacillus hunanensis* | GCM10008022_32200 | GCM10008022_32190 | CS | - |
| *Streptomyces zaomyceticus* | GCM10018791_64990 | GCM10018791_65010 | CS | - |
| *Paenibacillus polymyxa (strain E681)* | GE561_02376 | GE561_02377 | SSN/ CS | - |
| *Paenibacillus sp. OAE614* | GGC45_003599 | GGC45_003598 | CS | Soil -Oklahoma |
| *Streptomyces rimosus subsp. paromomycinus.* | GKJPGBOP_01912 | GKJPGBOP_01910 | SSN | Soil Colombia |
| *Streptomyces rimosus subsp. paromomycinus* | GKJPGBOP_01912 | GKJPGBOP_01910 | CS | soil |
| *Bacillus velezensis.* | GL331_06005 | GL331_05995 | SSN | - |
| *Streptomyces sp. SID1046* | GT352_39530 | GT352_39540 | CS | beetle |
| *Streptomyces sp. SID5471* | GTY89_25100 | GTY89_25090 | CS | - |
| *Bacillus amyloliquefaciens* | gutB1 | gabT1 | SSN | - |
| *Chitinophaga sp. H33E-04.* | GWR21_26060 | GWR21_26065 | SSN | - |
| *Bacillus subtilis subsp. spizizenii (strain TU-B-10)* | GYO_0348 | GYO_0346 | SSN | Water Michigan |
| *Paenibacillus sp. EKM208P* | H6F38_21085 | H6F38_21080 | CS | cantaloupe seeds |
| *Bacillus subtilis* | HC662_02040 | HC662_02020 | CS | *Arabidopsis thaliana* seedling |
| *Bacillus velezensis* | HCC49_00925 | HCC49_00915 | CS | *Juglans regia* leaf |
| *Paenibacillus sp. JGP012* | HNR77_004196 | HNR77_004197 | CS | - |
| *Paenibacillus taichungensis* | HP548_17685 | HP548_17680 | CS | soil- Taiwan |
| *Bacillus tequilensis* | HPX95_16195 | HPX95_16205 | CS | stem from dragon fruit tree |
| *Bacillus pacificus* | HQH96_20110 | HQH96_20100 | CS | soil from tomato plant |
| *Bacillus velezensis* | | HQK18_15155 | HQK18_15165 | CS |
| *Bacillus velezensis* | HRF58_18595 | HRF58_18585 | CS | Camel rumen fluid |
| *Bacillus sp. EKM213B* | HUN09_20660 | HUN09_20670 | CS | cantaloupe seeds |
| *Bacillus velezensis* | HUW30_00810 | HUW30_00800 | CS | stingray |
| *Paenibacillus sp. E222* | HW560_18280 | HW560_18285 | CS | Epichloe |
| *Bacillus velezensis* | HYH26_13030 | HYH26_13020 | CS | Soil - Russia |
| *Bacillus cereus BAG1X1-1.* | ICC_05063 | ICC_05065 | SSN | - |
| *Bacillus cereus BAG1X2-1.* | ICI_05398 | ICI_05396 | SSN | - |
| *Bacillus cereus BAG1X2-2.* | ICK_05452 | ICK_05450 | SSN | - |
| *Bacillus cereus BAG1X2-3.* | ICM_05871 | ICM_05869 | SSN | - |
| *Bacillus cereus BAG2O-1.* | ICO_05457 | ICO_05459 | SSN | - |
| *Bacillus sp. 1021* | ICW23_14560 | ICW23_14550 | CS | rice root |
| *Paenibacillus sp. CFBP 13594* | IFU39_01945 | IFU39_01950 | CS | Phaseolus vulgaris seed |
| *Bacillus cereus VD133.* | IIU_06842 | IIU_06844 | SSN/ CS | - |
| *Bacillus cereus VD169.* | IKA_05463 | IKA_05465 | SSN/ CS | - |
| *Paenibacillus cineris* | J21TS7_41110 | J21TS7_41100 | CS | honey- Japan |
| *Paenibacillus cineris* | J43TS9_00750 | J43TS9_00760 | CS | honey |
| *Cutibacterium acnes JCM 18909.* | JCM18909_3404 | JCM18909_3402 | SSN | - |
| *Bacillus cereus group sp. N21* | JDS96_24585 | JDS96_24595 | CS | Soil - Turkey |
| *Bacillus atrophaeus* | JEM62_17570 | JEM62_17580 | CS | soil- Antarctica |
| *Paenibacillus sp. MAHUQ-46* | JFN88_01590 | JFN88_01595 | CS | Soil - South Korea |
| *Bacillus sp. RHF6* | JMN15_18785 | JMN15_18795 | CS | Shoreline Rhizosphere |
| *Bacillus sp. RHFS18* | JMN16_09150 | JMN16_09160 | CS | Shoreline Rhizosphere |
| *Paenibacillus sp. JNUCC-31* | JNUCC31_01880 | JNUCC31_01875 | CS | Soil South Korea |
| *Paenibacillus nicotianae* | JOD35_002751 | JOD35_RS13665 | CS | - |
| *Paenibacillus sp. PvR133* | JOE49_003556 | JOE49_003557 | CS | Soil - Michigan |
| *Archangium violaceum* | JRI60_01010 | JRI60_01000 | CS | soil - China |
| *Streptomyces nojiriensis* | JYK04_07092 | JYK04_07090 | CS | - |
| *Paenibacillus polymyxa* | JYU28_10730 | JYU28_10725 | CS | commercial industrial hemp seed oil |
| *Paenibacillus mucilaginosus (strain KNP414)* | KNP414_01889 | KNP414_01888 | SSN/ CS | - |
| *Photorhabdus luminescens* | KS18_23560 | KS18_23565 | SSN | - |
| *Pedobacter cryoconitis.* | LY11_02602 | LY11_02601 | SSN | Austria |
| *Ruminiclostridium sufflavum DSM 19573.* | LY28_01664 | LY28_01665 | SSN | - |
| *Cosenzaea myxofaciens ATCC 19692.* | M983_2875 | M983_2876 | SSN | normal human and animal intestinal flora |
| *Photorhabdus khanii NC19.* | MB27_00240 | MB27_00235 | SSN | - |
| *Photorhabdus temperata subsp. temperata Meg1.* | MEG1DRAFT_02967 | MEG1DRAFT_02968 | SSN | - |
| *Myxococcus fulvus.* | MFU01_64050 | MFU01_64040 | SSN | - |
| *Bacillus amyloliquefaciens Y2.* | MUS_0169 | MUS_0167 | SSN/ CS | Wheat Rhizosphere |
| *Bacillus sp. 5B6.* | MY7_0007 | MY7_0005 | SSN/ CS | *Prunus avium*(sweet cherry tree) leaf South Korea |
| *Bacillus sp. JS.* | MY9_0162 | MY9_0160 | SSN/ CS | Rhizosphere of plants |
| *Paenibacillus polymyxa* | NCTC10343_02577 | NCTC10343_02578 | SSN | - |
| *Bacillus amyloliquefaciens EGD-AQ14.* | O205_16430 | O205_16440 | SSN |  |
| *Streptomyces chrestomyceticus JCM 4735* | OEIGOIKO_01846 | OEIGOIKO_01844 | CS | soil |
| *Actinobacteria bacterium OK074.* | OK074_7062 | OK074_7060 | SSN/ CS | Endosphere of plant *Populus trichocarpa* |
| *Paenibacillus sp. MAEPY2.* | P364_0112325 | P364_0112320 | SSN/ CS | soil |
| *Paenibacillus sp. MAEPY2.* | P364_0132450 | P364_0132445 | SSN | soil |
| *Paenibacillus sp. GM1FR.* | PAEAM_19310 | PAEAM_19320 | SSN/ CS | - |
| *Paenibacillus sp. GM1FR.* | PAEAM_29110 | PAEAM_29100 | SSN | *Festuca rubra* (grass) Germany |
| *Paenibacillus amylolyticus.* | PAHA3_1643 | PAHA3_1644 | SSN | - |
| *Cutibacterium acnes HL201PA1.* | PAST3_06327 | PAST3_06317 | SSN/CS | Host: *Homo sapiens* |
| *Photorhabdus asymbiotica subsp. asymbiotica (strain ATCC 43949 / 3105-77)* | PAU_00382 | PAU_00381 | SSN | - |
| *Photorhabdus namnaonensis.* | Phpb_01420 | Phpb_01419 | SSN | Entomopathogenic *Heterorhabditis baujardi* |
| *Paenibacillus illinoisensis.* | PIL02S_05729 | PIL02S_05730 | SSN | - |
| *Photorhabdus laumondii subsp. laumondii (strain DSM 15139 / CIP 105565 /TT01)* | PLU_RS02315 | PLU_RS02310 | SSN | Nematode |
| *Paenibacillus mucilaginosus 3016.* | PM3016_2175 | PM3016_2174 | SSN/ CS | Rhizosphere soil |
| *Photorhabdus australis.* | Ppb6_02665 | Ppb6_02666 | SSN | Nematodes |
| *Paenibacillus polymyxa* | PPOLYM_03986 | PPOLYM_03985 | SSN | - |
| *Paenibacillus polymyxa (strain SC2)* | PPSC2_12150 | PPSC2_12155 | SSN/ CS | Rhizosphere |
| *Paenibacillus polymyxa SQR-21* | PPSQR21_02430 | PPSQR21_024240 | CS |  |
| *Paenibacillus polymyxa* | PPYC2_14045 | PPYC2_14040 | CS | Rhizosphere of tobacco |
| *Phytohabitans rumicis* | Prum_101850 | Prum_101830 | CS | - |
| *Photorhabdus khanii NC19.* | PTE_02081 | PTE_02082 | SSN | Soil - North Carolina |
| *Paenibacillus nuruki.* | PTI45_01412 | PTI45_01411 | SSN/ CS | Korean traditional nuruk |
| *Bacillus atrophaeus* | RA13_00780 | RA13_00790 | CS | cotton root |
| *Bacillus velezensis (strain DSM 23117 / BGSC 10A6 / FZB42)* | RBAM_002060 | RBAM_002040 | SSN/ CS | Plant-pathogen infested soil Germany; Known producer of DNJ |
| *Bacillus velezensis UCMB5033.* | RBAU_0176 | RBAU_0174 | SSN | Plant associated |
| *Bacillus atrophaeus* | S101359_00194 | S101359_00192 | CS | food - South Korea |
| *Photorhabdus luminescens* | SAMN02982990_04307 | SAMN02982990_04308 | SSN | - |
| *Paenibacillus sp. 276b.* | SAMN03159332_1525 | SAMN03159332_1524 | SSN | - |
| *Paenibacillus sp. 276b.* | SAMN03159332_2658 | SAMN03159332_2657 | SSN | - |
| *Paenibacillus algorifonticola.* | SAMN04487969_102337 | SAMN04487969_102338 | SSN |  |
| *Chitinophaga filiformis* | SAMN04488121_103234 | SAMN04488121_103235 | SSN | - |
| *Bacillus sp. 103mf.* | SAMN04488145_1269 | SAMN04488145_1267 | SSN | Plant Root |
| *Bacillus sp. 491mf.* | SAMN04488168_12213 | SAMN04488168_12211 | SSN/ CS | - |
| *Chitinophaga rupis.* | SAMN04488505_1021137 | SAMN04488505_1021138 | SSN | soil |
| *Clostridium gasigenes.* | SAMN04488529_101211 | SAMN04488529_101210 | SSN | Spoiled foods |
| *Bacillus sp. 71mf* | SAMN04488574_14312 | SAMN04488574_14314 | CS | - |
| *Actinoplanes derwentensis.* | SAMN04489716_4921 | SAMN04489716_4922 | SSN | Soil |
| *Chitinophaga sp. CF418.* | SAMN05216311_103474 | SAMN05216311_103473 | SSN | Populus root |
| *Sinosporangium album.* | SAMN05421505_11027 | SAMN05421505_11029 | SSN/ CS | soil |
| *Actinoplanes philippinensis.* | SAMN05421541_102253 | SAMN05421541_102254 | SSN | - |
| *Xenorhabdus japonica.* | SAMN05421579_12720 | SAMN05421579_12719 | SSN | Entomopathogenic nematode |
| *Xenorhabdus mauleonii.* | SAMN05421680_12624 | SAMN05421680_12623 | SSN | Entomopathogenic nematode |
| *Xenorhabdus koppenhoeferi.* | SAMN05421784_12423 | SAMN05421784_12422 | SSN | Entomopathogenic nematode |
| *Mucilaginibacter lappiensis.* | SAMN05421821_10412 | SAMN05421821_10411 | SSN | Lichen and soil |
| *Streptomyces sp. 2314.4.* | SAMN05428943_0992 | SAMN05428943_0990 | SSN | - |
| *Chitinophaga sp. YR627.* | SAMN05428949_2386 | SAMN05428949_2385 | SSN | Populus root |
| *Paenibacillus sp. OK060* | SAMN05428961_102637 | SAMN05428961_102636 | CS | - |
| *Paenibacillus sp. CF095.* | SAMN05428987_1408 | SAMN05428987_1409 | SSN/ CS | - |
| *Paenibacillus sp. CF095.* | SAMN05428987_2246 | SAMN05428987_2247 | SSN | - |
| *Chitinophaga sp. YR573.* | SAMN05428988_5440 | SAMN05428988_5439 | SSN | Populus root |
| *Pedobacter hartonius.* | SAMN05443550_105340 | SAMN05443550_105341 | SSN/ CS | freshwater of the hard-water creek Westerhöfer Bach, North Germany. |
| *Marininema mesophilum.* | SAMN05444487_10325 | SAMN05444487_10323 | SSN / CS | Sediment South China Sea |
| *Bacillus sp. OK838.* | SAMN05444672_1368 | SAMN05444672_1366 | SSN | - |
| *Paenibacillus sp. OK076* | SAMN05518670_3146 | SAMN05518670_3145 | CS | - |
| *Chitinophaga sp. CF118.* | SAMN05518672_10347 | SAMN05518672_10346 | SSN | Populus root |
| *Paenibacillus sp. OK003.* | SAMN05518856_110240 | SAMN05518856_110241 | SSN/ CS | - |
| *[Clostridium] populeti.* | SAMN05661086_01768 | SAMN05661086_01767 | SSN | woody biomass digestor |
| *[Clostridium] populeti.* | SAMN05661086_03084 | SAMN05661086_03082 | SSN | woody biomass digestor |
| *Paenibacillus polysaccharolyticus.* | SAMN05720606_10216 | SAMN05720606_10215 | SSN | - |
| *Paenibacillus polysaccharolyticus.* | SAMN05720606_103176 | SAMN05720606_103175 | SSN/ CS | - |
| *Paenibacillus sp. RU4X.* | SAMN05880555_1530 | SAMN05880555_1531 | SSN | - |
| *Pedobacter westerhofensis.* | SAMN06265348_1106 | SAMN06265348_1105 | SSN/ CS | - |
| *Bacillus sp. Pc3* | SB24_08755 | SB24_08765 | CS | seawater |
| *Serratia proteamaculans.* | SPRA44_330036 | SPRA44_330035 | SSN | organic beef carpaccio |
| *Bacillus velezensis* | SRCM100731_03644 | SRCM100731_03642 | CS | Kochujang |
| *Bacillus amyloliquefaciens* | SRCM101294_03828 | SRCM101294_03830 | CS | Soil - South Korea |
| *Streptomyces rimosus subsp. rimosus (strain ATCC 10970 / DSM 40260 / JCM4667 / NRRL 2234)* | SRIM_11186 | SRIM_11176 | SSN/ CS | - |
| *Streptomyces afghaniensis 772.* | STAFG_8391 | STAFG_8392 | SSN | soil |
| *Streptomyces auratus AGR0001.* | SU9_29381 | SU9_29371 | SSN | soil - china |
| *Pedobacter lusitanus.* | TH53_07505 | TH53_07510 | SSN | soil |
| *Bacillus sp. TH008* | TH62_01230 | TH62_01220 | CS | Soil- Ohio |
| *Vibrio neptunius.* | TW84_21565 | TW84_21570 | SSN | - |
| *Bacillus subtilis* | UM89_12560 | UM89_12570 | CS | Soil - India |
| *Peribacillus simplex.* | UP17_10790 | UP17_10780 | SSN/ CS | sugar beet rhizosphere |
| *Paenibacillus etheri.* | UQ64_11740 | UQ64_11735 | SSN | - |
| *Streptomyces rimosus R6-500.* | V519_020255 | V519_020265 | SSN | - |
| *Paenibacillus sp. D9.* | VE23_04150 | VE23_04145 | SSN/ CS | soil |
| *Streptomyces malaysiense.* | VT52_021255 | VT52_021265 | SSN/ CS | Soil |
| *Photorhabdus thracensis.* | VY86_05755 | VY86_05760 | SSN | - |
| *Xenorhabdus beddingii.* | Xbed_02295 | Xbed_02296 | SSN | Entomopathogenic nematode |
| *Xenorhabdus bovienii str. feltiae Florida.* | XBFFL1_190022 | XBFFL1_190023 | SSN | - |
| *Xenorhabdus bovienii str. feltiae Moldova.* | XBFM1_80021 | XBFM1_80022 | SSN | nematode |
| *Xenorhabdus bovienii str. Intermedium.* | XBI1_1740025 | XBI1_1740024 | SSN | nematode |
| *Xenorhabdus bovienii (strain SS-2004)* | XBJ1_4037 | XBJ1_4038 | SSN | Nematode |
| *Xenorhabdus bovienii str. kraussei Becker Underwood.* | XBKB1_370010 | XBKB1_370011 | SSN | Nematode |
| *Xenorhabdus bovienii str. kraussei Quebec.* | XBKQ1_220021 | XBKQ1_220022 | SSN | Nematode |
| *Xenorhabdus bovienii str. oregonense.* | XBO1_170020 | XBO1_170021 | SSN | Nematode |
| *Xenorhabdus bovienii str. puntauvense.* | XBP1_110022 | XBP1_110023 | SSN | Nematode |
| *Xenorhabdus budapestensis.* | Xbud_00960 | Xbud_00959 | SSN | Nematode |
| *Xenorhabdus bovienii.* | XBW1_0423 | XBW1_0422 | SSN | Nematode |
| *Xenorhabdus cabanillasii JM26.* | XCR1_1330023 | XCR1_1330024 | SSN | Nematode |
| *Xenorhabdus doucetiae.* | XDD1_3627 | XDD1_3628 | SSN | Nematode |
| *Xenorhabdus eapokensis.* | Xedl_02204 | Xedl_02203 | SSN | Nematode |
| *Xenorhabdus ehlersii.* | Xehl_00218 | Xehl_00219 | SSN | Nematode |
| *Xenorhabdus thuongxuanensis.* | Xentx_02834 | Xentx_02833 | SSN | Nematode |
| *Xenorhabdus hominickii.* | Xhom_01060 | Xhom_01059 | SSN | Nematode |
| *Xenorhabdus ishibashii.* | Xish_01931 | Xish_01930 | SSN | Nematode |
| *Xenorhabdus kozodoii.* | Xkoz_00753 | Xkoz_00754 | SSN | Nematode |
| *Xenorhabdus miraniensis.* | Xmir_01935 | Xmir_01934 | SSN | Nematode |
| *Xenorhabdus nematophila F1.* | XNC3_2600007 | XNC3_2600008 | SSN | - |
| *Xenorhabdus nematophila str. Websteri.* | XNW1_1520007 | XNW1_1520008 | SSN | Nematode |
| *Xenorhabdus poinarii G6.* | XPG1_3156 | XPG1_3157 | SSN | Nematode |
| *Xenorhabdus vietnamensis.* | Xvie_02778 | Xvie_02777 | SSN | Nematode |
| *Xenorhabdus nematophila (strain ATCC 19061 / DSM 3370 / LMG 1036 / NCIB9965 / AN6)* | XNC1_4281 | XNC1_4282 | SSN | Nematode |

Table S1
